# Species diversity and molecular characterization of *Alternaria* section *Alternaria* isolates collected mainly from cereal crops in Canada

**DOI:** 10.3389/fmicb.2023.1194911

**Published:** 2023-05-25

**Authors:** Jeremy R. Dettman, Quinn A. Eggertson, Natalie E. Kim

**Affiliations:** Agriculture and Agri-Food Canada, Ottawa Research and Development Centre, Ottawa, ON, Canada

**Keywords:** *Alternaria alternata*, *A. arborescens*, *ASA-10*, *ASA-19*, *RPB2*, species complex

## Abstract

*Alternaria* is often one on the most abundant fungal genera recovered from a wide array of plant hosts and environmental substrates. Many species within the sub-generic *Alternaria* section *Alternaria* are common plant pathogens that cause pre-harvest losses due to reduced productivity and post-harvest losses due to spoilage and contamination with mycotoxins. As certain species of *Alternaria* may have distinct mycotoxin profiles, and very broad host ranges, understanding the distribution of species by geography and host is critical for disease prediction, toxicological risk assessment, and guiding regulatory decisions. In two previous reports, we performed phylogenomic analyses to identify highly informative molecular markers for *Alternaria* section *Alternaria*, and validated their diagnostic ability. Here, we perform molecular characterization of 558 section *Alternaria* strains, collected from 64 host genera in 12 countries, using two of these section-specific loci (*ASA-10* and *ASA-19*) along with the RNA polymerase II second largest subunit (*rpb2*) gene. The majority of strains (57.4%) originated from various cereal crops in Canada, which formed the main focus of our study. Phylogenetic analyses were used to classify strains into section *Alternaria* species/lineages, demonstrating that the most common species on Canadian cereal crops are *Alternaria alternata* and *A. arborescens.* Further population genetic analyses were consistent with *A. alternata* being a widely distributed species with relatively low levels of geographic isolation (i.e., Canadian isolates did not form distinct clades when compared to other regions). Our expanded sampling of *A. arborescens* has greatly increased the known diversity of this group, with *A. arborescens* isolates forming at least three distinct phylogenetic lineages. Proportionally, *A. arborescens* is more prevalent in Eastern Canada than in Western Canada. Sequence analyses, putative hybrids, and mating-type distributions provided some evidence for recombination events, both within and between species. There was little evidence for associations between hosts and genetic haplotypes of *A. alternata* or *A. arborescens*.

## Introduction

Members of the widespread fungal genus *Alternaria* are commonly found in a broad diversity of niches and environmental substrates (Lawrence et al., 2013; Woudenberg et al., 2013). Several recent surveys of various plant microbiomes have demonstrated that *Alternaria* is often one on the most abundant fungal genera recovered (e.g., Johnston-Monje et al., 2021; Latz et al., 2021; Morales Moreira et al., 2021; Hafez et al., 2022; Yan et al., 2022). While some species may be saprobes or harmless endophytes, many other species are economically significant pathogens of important agricultural crops (Thomma, 2003). Hundreds of different crops are affected by *Alternaria* diseases, with infections occurring on a wide range of plant structures such as stems, leaves, seeds, and fruits (Rotem, 1994; Woudenberg et al., 2013; United States Department of Agriculture National Fungus Collections Fungus-Host Dataset, 2021). Several prominent plant pathogenic species fall within the sub-generic *Alternaria* section *Alternaria* (Lawrence et al., 2013; Woudenberg et al., 2015a), including one of the most widely distributed and commonly found species, *A. alternata*.

In addition to pre-harvest problems that reduce primary production, *Alternaria* can cause further post-harvest losses via food spoilage or synthesis of dangerous mycotoxins (e.g., alternariol and tenuazonic acid) that can contaminate food and consumer end-products (Mujahid et al., 2020; Aichinger et al., 2021). International regulatory agencies such as the European Food Safety Authority have highlighted the public health risks of *Alternaria* mycotoxins, particularly in grain and grain-based products, and have concluded that much more targeted research on toxicity and occurrence is needed (EFSA, 2011; EFSA, 2016). In Canada, surveys of main cereal crops have shown that *Alternaria*-associated toxins are commonly detected (Tittlemier et al., 2019; Kelman et al., 2020), however, the taxonomic identity of the causal *Alternaria* species often remains unclear.

As certain species of *Alternaria* may have distinct and unique mycotoxin profiles (Meena et al., 2017; Tralamazza et al., 2018), reliable methods of detection and identification based on a stable biosystematics framework are critical for toxicological risk assessment and guiding regulatory and quarantine decisions. Confidently identifying species within *Alternaria* section *Alternaria* is challenging for two reasons: (1) “diagnostic” morphological characteristics tend to be phenotypically plastic and overlap between species and (2) molecular markers typically used in fungal phylogenetics are not sufficiently informative to distinguish among section *Alternaria* species (Andrew et al., 2009; Armitage et al., 2015; Woudenberg et al., 2015a; Armitage et al., 2020). To address this problem, we recently performed comprehensive phylogenomic analyses of section *Alternaria* and scanned genomes for genes that were top candidates for development into informative and phylogenetically accurate molecular markers (Dettman and Eggertson, 2021). In a subsequent report, we developed locus-specific primer sets for three of these candidate markers and demonstrated they were able to consistently classify section *Alternaria* strains to species/lineage (Dettman and Eggertson, 2022).

Here, we assess the species diversity and molecular variation of a large sample of *Alternaria* section *Alternaria* strains by analyzing sequences from two of our recently developed section-specific markers (*ASA-10* and *ASA-19*), along with the RNA polymerase II second largest subunit (*rpb2*) gene. Sequence data were generated for 385 newly characterized strains and combined with corresponding sequences from 173 other section *Alternaria* strains that were examined previously or available in public sequence repositories. In total, our final data matrix consisted of 558 strains and three loci, representing, to our knowledge, the largest multi-locus dataset for section *Alternaria* to date. Although a wide diversity of isolation sources were represented in our sample (e.g., 64 host genera, 12 countries), the majority of strains (320, 57.4%) originated from cereal crops in Canada, the main focus of our study. We perform phylogenetic analyses to classify strains into species/lineages within section *Alternaria* and find that the most prevalent taxon in Canada is *A. alternata*, followed by *A. arborescens*. Further detailed analyses were performed to examine patterns of within-lineage variation, association of genetic haplotypes with host and geography, and evidence of recombination.

## Materials and methods

### Fungal strains

The 385 newly characterized section *Alternaria* strains (Supplementary Table S1) were obtained from mycological research collections held by Agriculture & Agri-Food Canada (AAFC) in Ottawa, Canada. The 99 strains with KAS or SLOAN prefixes are from the collection of Keith Seifert, whereas the 286 strains with DET prefixes are part the Dettman laboratory collection. Details of geographic source, substrate, and year of collection are provided in Supplementary Table S1. To maximize the representation of non-Canadian strains, we included the widest possible range of existing section *Alternaria* strains we could obtain from AAFC culture collections (and genome sequence databases – see below). For geographic source, Eastern Canada was defined as Ontario and all provinces to the east, whereas Western Canada was defined a Manitoba and all provinces to the west.

### DNA extraction

Fungal strains were grown on potato dextrose agar (potato extract, 4 g/L; dextrose, 20 g/L; agar 15 g/L) at 25°C and tissue was harvested into lysis buffer and 25 uL of Proteinase K in sterile 2.0 mL o-ring tubes containing 0.25 mL of 1 mm zirconia beads (BioSpec Products, Bartlesville, OK, United States). Tissue was homogenized at 6000 rpm for 40 s using a Precellys 24 homogenizer (Bertin Instruments, Montigny-le-Bretonneux, France). Genomic DNA was then extracted using the Macherey-Nagel NucleoMag 96 Trace kit (Macherey Nagel, Düren, Germany) and a KingFisher Flex magnetic particle processor (Thermo Fisher Scientific, Waltham, MA, United States) following the manufacturer’s suggested protocols.

### PCR and Sanger sequencing

PCR protocols, sequencing methods, and primer sequences were as described in Dettman and Eggertson (2022). Thermocycler profiles were as follows: initial denaturation at 94°C for 2 min, followed by 35 cycles of 94°C for 30 s, annealing for 30 s (58°C for *ASA-10*, 55°C for *ASA-19*, 53°C for *rpb2*), extension at 72°C for 1 min, with a final extension at 72°C for 10 min. In some cases, 45 PCR cycles were performed for *ASA-10* amplification. When using *rpb2* primers from Liu et al. (1999), denaturation was at 95°C for 2 min, followed by 40 cycles of 95°C for 1 min, annealing at 55°C for 2 min, extension at 72°C for 2 min, with a final extension at 72°C for 10 min. Pre-sequencing amplification was done with BigDye™ Terminator v3.1 Cycle Sequencing Kits (Applied Biosystems, Waltham, MA, USA) with an initial denaturation of 95°C for 3 min, followed by 40 cycles of 95°C for 30 s, annealing for 20 s (58°C for *ASA-10*, 55°C for *ASA-19*, and 50°C for all *rpb2*), with an extension for 60°C for 2 min. DNA sequence data were generated using an 3130xl Genetic Analyzer (Applied Biosystems) in the Molecular Technologies Laboratory (Agriculture & Agri-Food Canada). Geneious Prime (2022.01.01; Biomatters, Auckland, New Zealand) was used to visualize sequence data and determine consensus sequences. Mating-type idiomorphs were determined by multiplex PCR amplification with two sets of idiomorph-specific primers described by Gannibal and Kazartsev (2013). Primers for MAT1-1 were Amat1cF (5’-AAGCARATGGTTCARTTGTTCC-3′) and Amat1R (5’-GACCAGGCTTTCGYCATC-3′) and primers for MAT1-2 were ATENF1 (5’-AGCCCTTCTCACTTGCACTG-3′) and MCHMG2 (5’-CTGGGRGTRTACTTGTAGTCRGG-3′). Thermocycler profiles were as follows: initial denaturation of 94°C for 3 min, followed by 30 cycles of 92°C for 50 s, annealing at 55°C for 50 s, with an extension for 72°C for 60 s. PCR products were electrophoresed on agarose gels and scored for the idiomorph-specific product sizes (MAT1-1 ~ 300 bp; MAT1-2 ~ 500 bp).

### Previously released sequence data

To build on our previous work, we include here 86 section *Alternaria* strains from Dettman and Eggertson (2022) for which sequences for all three loci (*ASA-10*, *ASA-19*, and *rpb2*) were available. Thirty-nine of these strains were sequenced via Sanger methodology (accession numbers in Supplementary Table S2) and locus sequences for the remaining 47 strains were extracted from whole genome assemblies (accession numbers in Supplementary Table S3) as described in Dettman and Eggertson (2022). Although much of this information can be found in our previous reports, we include it here to allow for ease of cross-comparison of strain metadata. Locus sequences were also extracted from an additional 87 section *Alternaria* genome assemblies that became publicly available after our last study, and from *A. solani* (section *Porri*) strain HWC-168-2012p to represent an outgroup (Supplementary Table S3). We incorporated all genomes from section *Alternaria* strains that were available at project initiation (October 1, 2022).

### Sequence analyses and phylogenetic methods

Sequences were aligned with MUSCLE (3.8; Edgar, 2004) using default parameters, then alignments were verified by visual inspection and statistics were calculated with AMAS (Borowiec, 2016). Selection of substitution models and construction of maximum likelihood (ML) phylogenies were performed with IQ-TREE (2.0; Nguyen et al., 2015). The MODELFINDER module was used to determine the best substitution model for each locus and the optimal partitioning scheme for multi-locus datasets (−m MFP + MERGE). ML trees were constructed under best-fit substitution models and partitions, with branch support being assessed with 1,000 non-parametric bootstrap replicates (−b 1,000). MrBayes (3.2.6; Ronquist and Huelsenbeck, 2003) was used for Bayesian phylogenetic inference of the combined dataset with a GTR substitution model, gamma-distributed rate variation across sites, and a proportion of invariable sites (nst = 6, rates = invgamma, ploidy = haploid). Default priors were used for all analyses, the starting trees were random (starttree = random), and each locus was treated as a separate partition with independent parameter estimation. Two runs with four chains each were run for two million generations (ngen = 2,000,000) with a sampling frequency of every 100 generations (samplefreq = 100), and the first 25% of sampled trees were discarded as burn-in. Nucleotide diversity was calculated with MEGA (11.0.13; Tamura et al., 2021). POPART (1.7.2; Leigh and Bryant, 2015) was used to generate and display TCS haplotype networks. Recombination analyses were performed with RDP (4.101; Martin et al., 2015) using six different detection methods: RDP, GENECONV, BootScan, MaxChi, Chimaera, SiScan and 3Seq. The value of p was set to 0.05 with Bonferroni correction applied, breakpoints were polished, and only events detected by two or more methods of detection were reported.

## Results

### *Alternaria* section *Alternaria* strain sampling

**Figure 1:**
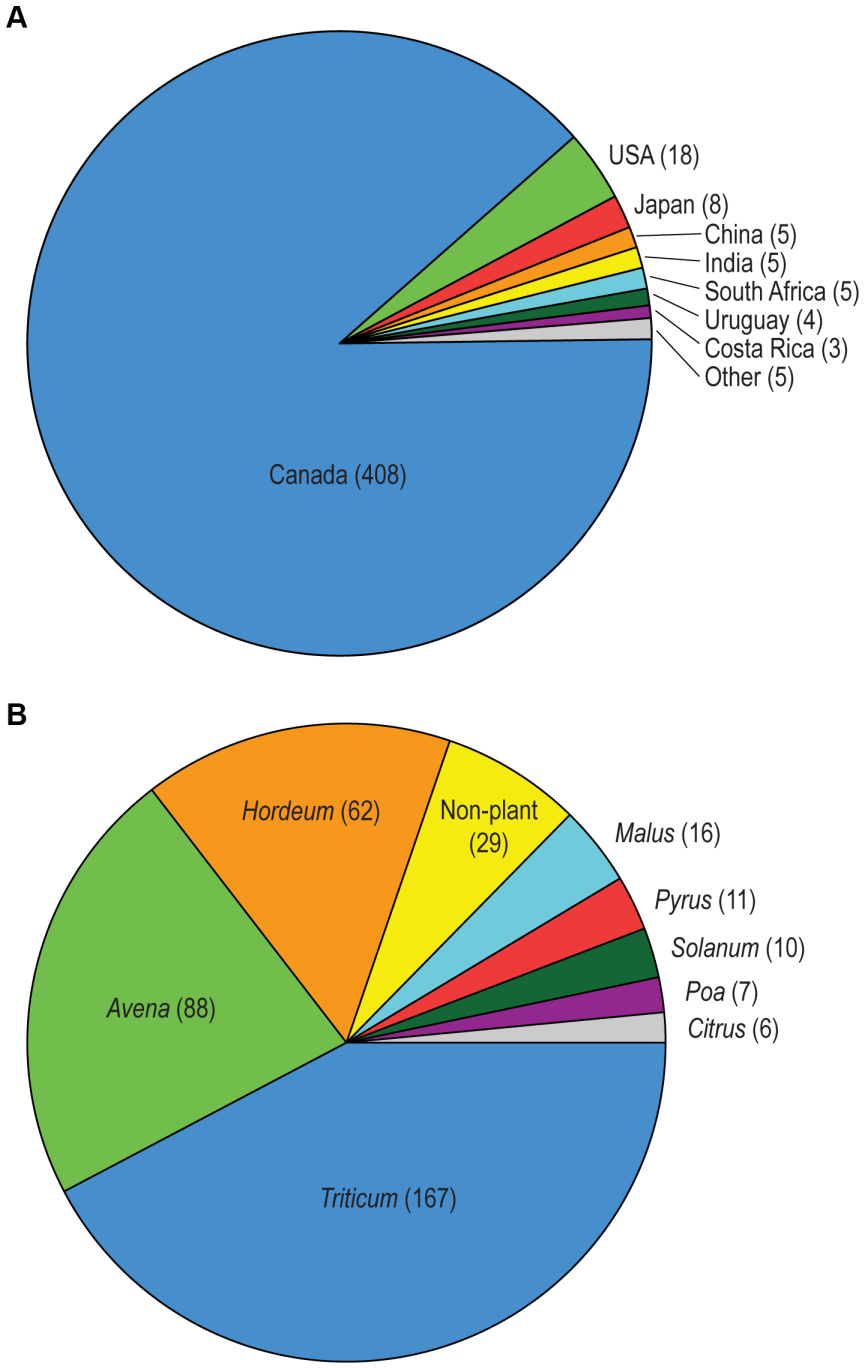
**(A)** Geographic sources of the *Alternaria* section *Alternaria* strains, with strain counts in parentheses. The “Other” category includes Germany, South Korea, Malawi, and the United Kingdom combined. The 97 strains with genome assemblies that lacked a declared geographic source are not included. **(B)** Most common substrate/host sources for the *Alternaria* section *Alternaria* strains, with strain counts in parentheses. Only categories with >5 samples are shown. Substrate sources of all strains can be found in Supplementary Tables S1–3.

We investigated the molecular variation and species diversity of a total of 558 *Alternaria* section *Alternaria* strains. The majority (429, 76.9%) are live, cultured strains from Agriculture and Agri-Food Canada mycological research collections and the remaining (129, 23.1%) strains are represented by sequences extracted from their genome assemblies. Overall, 84.6% of these strains (472; 385 live strains and 87 genomes) were not included in any of our previous studies. The geographic and substrate/host diversity of the strain sample was quite broad, with isolation sources in 12 countries from 64 plant host genera, as well as non-plant substrates such as soil and indoor biomes. As the central focus of our study, the majority of strains (320, 57.4%) originated from Canadian cereal crops such as wheat, oats, and barley (*Triticum*, *Avena*, and *Hordeum*, respectively; Figure 1).

### Molecular markers

For molecular characterization, we chose two of our recently developed markers, *ASA-10* and *ASA-19*, which are diagnostic for the main phylogenetic lineages within *Alternaria* section *Alternaria*. *ASA-10* encodes an F-box-domain-containing protein and *ASA-19* encodes a histone-fold-containing protein. For comparison to a more commonly used molecular marker, the housekeeping gene RNA polymerase II second largest subunit (*rpb2*) was also included. Genomic DNA was extracted and these three loci were sequenced from the 385 newly characterized section *Alternaria* strains using standard Sanger methodologies. These data were combined with corresponding sequences from 173 other strains obtained in our previous work or extracted from genome assemblies. For the sake of data matrix completeness, we included only strains with sequence data for all three loci. Summary statistics for the final alignments for each locus are shown in Table 1. Consistent with previous results, the *ASA* loci were highly variable and had greater proportions of phylogenetically informative sites than *rpb2*.

**Table 1:** Summary information for single-locus alignments.

Locus	Alignment length	Number of variable sites	Proportion variable sites	Number of informative sites	Proportion informative sites
*ASA-10*	379	97	0.256	41	0.108
*ASA-19*	547	111	0.203	59	0.108
*rpb2*	870	117	0.134	61	0.070

### Lineage assignment via phylogenetic analyses

**Figure 2:**
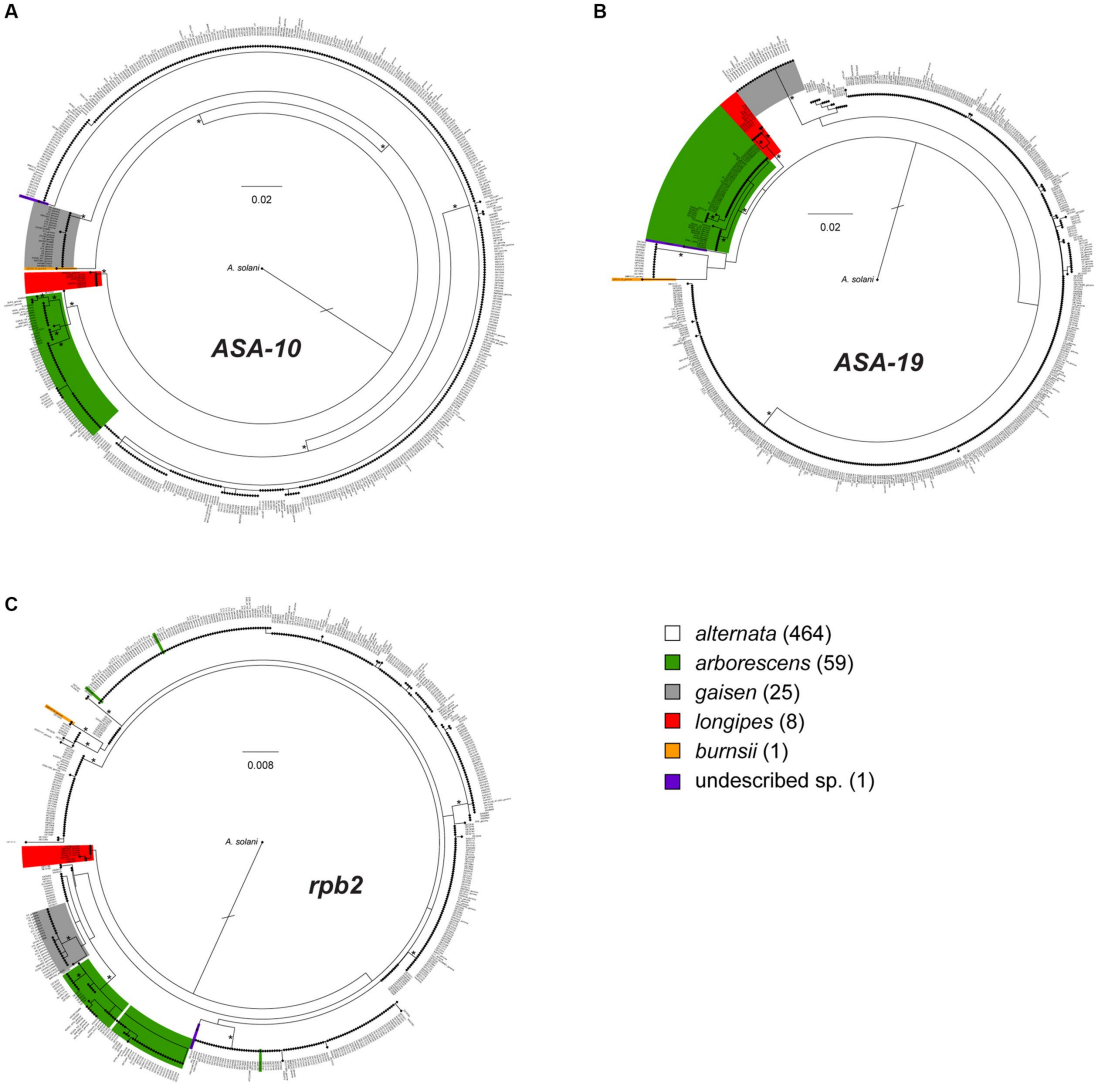
Maximum likelihood phylograms constructed from the single-locus alignments for **(A)** *ASA-10*, **(B)** *ASA-19*, and **(C)** *rpb2* (RNA polymerase II second largest subunit). The 559 taxa are color-coded to indicate placement within phylogenetic lineages, as determined by combined analyses of the three loci together (see Figure 3). In the color legend, numbers of strains within grouping are shown in parentheses. The actual length of the branch leading to *A. solani* (outgroup) is twice as long as shown. Major branches with ≥70% bootstrap percentages are marked by an asterisk.

The full, 559-taxon maximum likelihood (ML) trees constructed from each of the three loci are shown in Figure 2, with expanded, higher resolution versions shown Supplementary Figures S1–3. Using the 86 previously characterized section *Alternaria* strains as references, we used our phylogenetic approach (Dettman and Eggertson, 2022) to assign the 472 newly analyzed strains to a species/lineage. Consistent with previous work, the *ASA-10* and *ASA-19* loci both performed well at species diagnostics and recovering the main phylogenetic lineages within section *Alternaria*. While the main lineages tended to be monophyletic and well supported in the *ASA-10* and *ASA-19* trees, there were some exceptions. First, in the *ASA-10* tree, one strain of *A. alternata* (DET2202) was closely related and basal to the arborescens lineage (Supplementary Figure S1). Second, in the *ASA-19* tree, the alternata lineage was non-monophyletic and divided into three sub-clades, but with generally low levels of bootstrap support (Supplementary Figure S2). However, the three other main lineages (arborescens, gaisen, longipes) were monophyletic and well supported.

The *rpb2* locus recovered only two of the main lineages (longipes and gaisen) as monophyletic, but neither received significant bootstrap support. Furthermore, the alternata lineage was paraphyletic and broken in multiple clades, and the monotypic A*. burnsii* strain was not unique (Figure 2, Supplementary Figure S3). The most notable conflict with the *ASA* loci was the placement by *rpb2* of two alternata strains (DET2290 and DET2310) within the arborescens lineage, and the mixing of three arborescens strains (DET2289, DET2306, and DET2323) within alternata sub-clades. This *rpb2*-specific pattern suggests these strains are potential arborescens/alternata hybrids.

**Figure 3:**
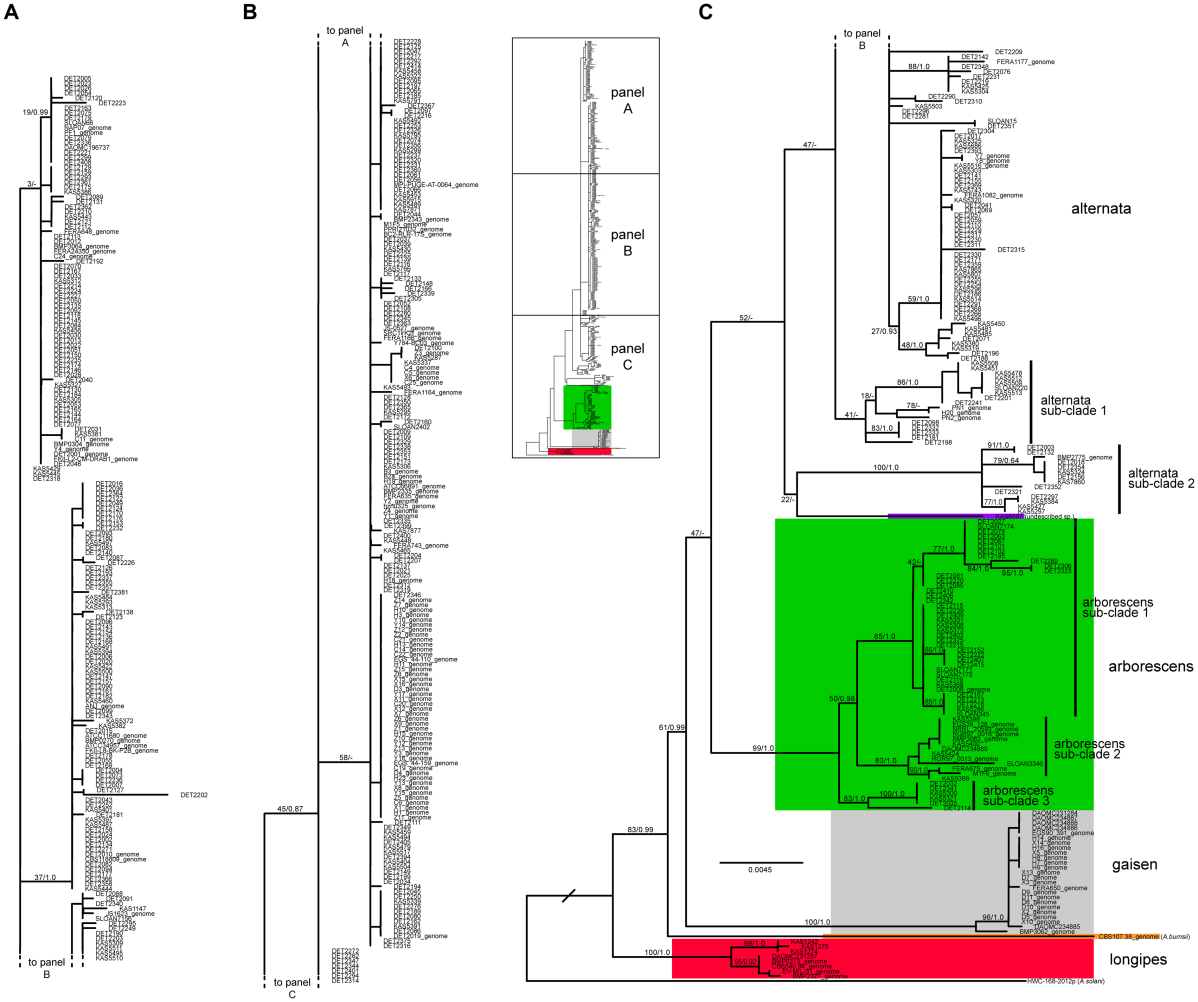
Maximum likelihood tree constructed from the three loci combined (*ASA-10*, *ASA-19*, and *rpb2*). The 559 taxa labels are color-coded to indicate placement within phylogenetic lineages. Maximum likelihood bootstrap percentages and Bayesian posterior probabilites are shown on major branches (MLBP/BPP). A dash indicates the branch was not present in the Bayesian consenses tree. Taxon labels followed by “_genome” indicate sequences that were extracted from genome assemblies. The actual length of the branch leading to *A. solani* (outgroup) is 5X as long as shown.

The three loci were combined and the ML tree constructed under the best-fit partition model is shown in Figure 3. As expected, the four main lineages were recovered, with significant support for the arborescens, gaisen, and longipes lineages. The large alternata clade (+ KAS6097, putative undescribed species) received only moderate support (52% ML bootstrap, not present in Bayesian consensus tree), likely due to the putative arborescens/alternata hybrids. When the strains with evidence of a hybrid origin were removed from the dataset and the analyses were performed again, that same clade received significant bootstrap support (88%, results not shown).

### Diversity by lineage/species

#### Alternata lineage

The vast majority (83%, *n* = 464) of our sample fell within the alternata lineage, which is currently represented by one large species, *A. alternata*. Combined analyses revealed the presence of a few sub-clades, most of which were not significantly supported. Two small, basal sub-clades (Figure 3) appeared to be fairly divergent from the rest of the species. Sub-clade 1 was composed of 17 strains whose placements varied slightly between the three loci. Sub-clade 2 was composed of 14 strains and was well supported, but this result was driven mainly by the fact that these strains all shared the same highly divergent allele at the *ASA-19* locus (Figure 2).

#### Arborescens lineage

**Figure 4:**
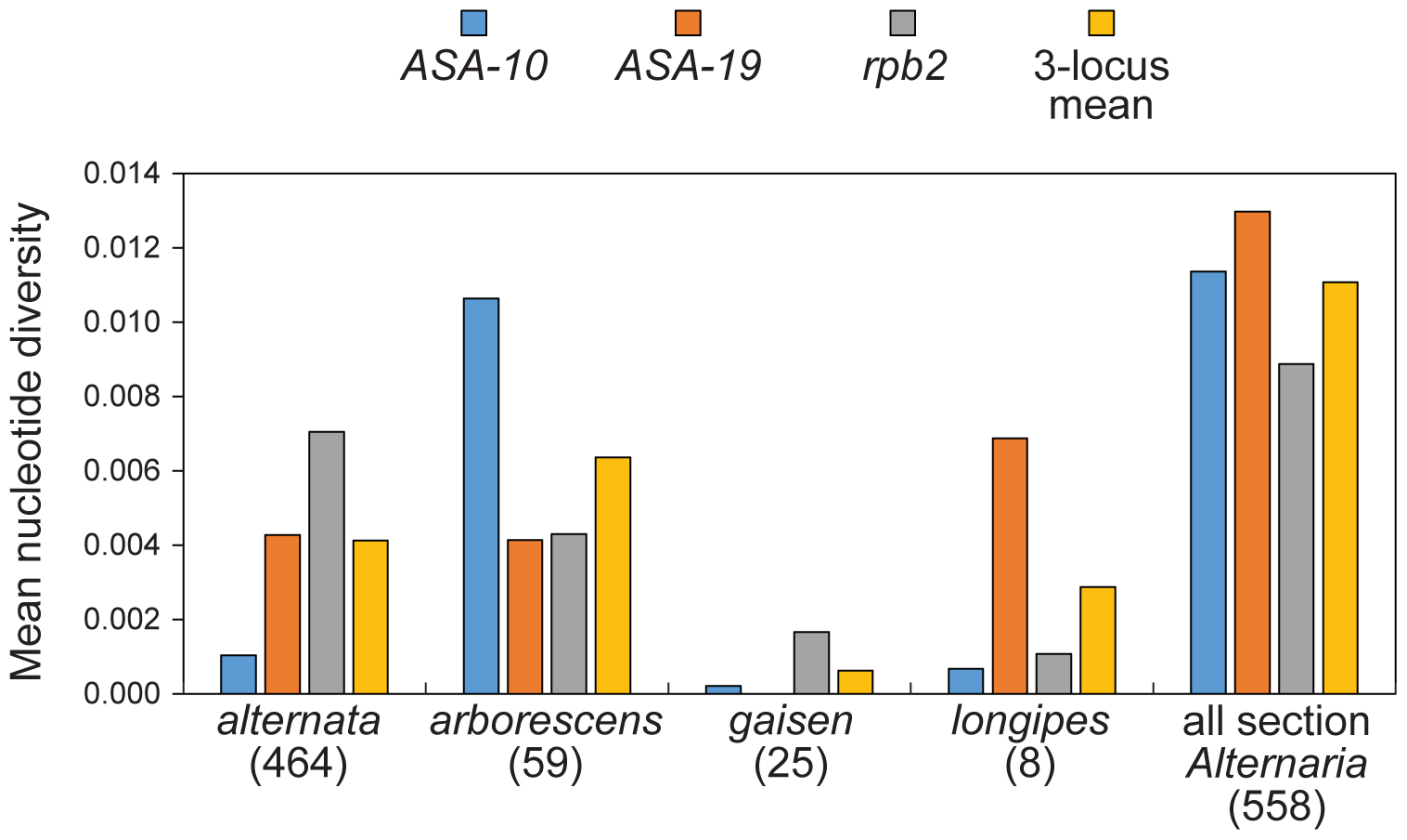
Mean nucleotide diversity values (Tamura-Nei model). Numbers in parentheses indicate number of strains within grouping. The “3-locus mean” is the unweighted mean of the mean values for the three separate loci.

The arborescens lineage was the second most common but it still accounted for only 10.6% (*n* = 59) of our sample. Despite the limited sample size, multiple sub-clades were identified within this lineage (sub-clades 1, 2, and 3) based on significant support in combined analyses (Figure 3) and concordant support from single-locus analyses (Supplementary Figures S1–3). Remarkably, all strains reported by other authors fall within sub-clade 2, indicating that our sampling of new strains has greatly expanded the known diversity of the arborescens lineage. Even though the sample size of the alternata lineage was 7.85X greater than the arborescens lineage, the mean nucleotide diversity of arborescens was 1.54X greater than that of alternata (0.0064 versus 0.0041, respectively; Figure 4).

#### Gaisen lineage

The gaisen lineage (=*A. gaisen*) was represented by 25 strains (4.5%) and displayed relatively low levels of molecular diversity. For example, all 25 strains shared the identical sequence at the *ASA-19* locus, despite *ASA-19* being most variable locus overall (Figure 2B, Figure 4).

#### Longipes lineage

Only 8 strains (1.4%) fell within the longipes lineage, which is composed of *A. longipes* and *A. gossypina*, typically associated with tobacco and tangerines, respectively. The strains isolated from tangerine did not form distinct groups in phylogenetic analyses, suggesting that *A. gossypina* may not be separate from *A. longipes*. Interestingly, our three KAS strains, all sampled from *Astronium* in Costa Rica, did form a distinct, well-supported clade.

#### Monotypic lineages

The single strain of *A. burnsii* (CBS107.38) and the undescribed species (KAS6097) each formed a distinct lineage in the *ASA-10* and *ASA-19* trees, however, their relationships with the other lineages were not consistent among loci.

To ensure that our potential within-lineage diversity did not actually correspond to other previously described species, we downloaded and analyzed reference *rpb2* sequences from the twelve section *Alternaria* species (*sensu* Woudenberg et al., 2015a), including the six that were not present in our sample: *A. alstroemeriae*, *A. betae-kenyensis*, *A. eichhorniae*, *A. iridaustralis*, *A. jacinthicola*, and *A. tomato*. Phylogenetic analyses (Supplementary Figure S4) confirmed our original findings and demonstrated that none of the newly characterized strains belonged to any of these six other species.

### Population genetic analyses: geographic source

**Figure 5:**
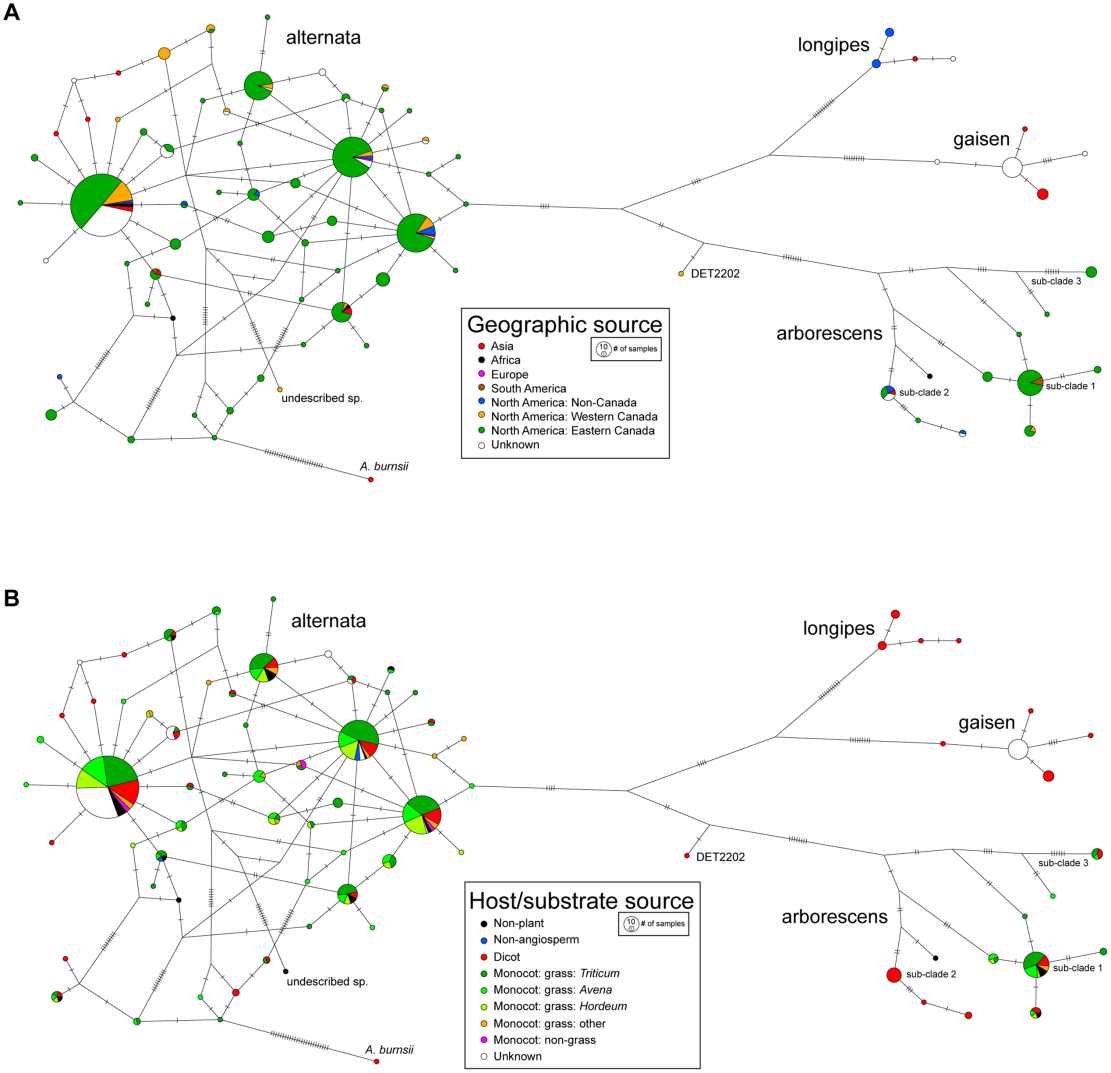
TCS (statistical parsimony) networks for multi-locus haplotypes. Mutations are indicated on branches as hatch marks. Node circle size is proportional to number of strains with the haplotype. Mapped onto the networks are **(A)** geographic source and **(B)** host/substrate source.

A TCS (statistical parsimony) network was constructed from the multi-locus haplotype data and trait states for geography were mapped onto the network (Figure 5A). Due to the large imbalance in sample size by continent, we divided the intensively sampled North America into three regions: Western Canada, Eastern Canada, and non-Canada. For the lineages with sufficient sampling, the general pattern was a lack of geographic partitioning of haplotypes. For example, the most frequently recovered haplotype of *A. alternata* was found in Asia, Africa, Europe, and all three regions of North America. The arborescens lineage was found in Asia, Africa, South America and North America, but smaller sub-clades were restricted to certain geographic areas (e.g., sub-clade 3 in Eastern Canada only). *A. gaisen* strains were not found outside of Asia, which was expected because this species is typically associated with Asian pear.

Within Canada, some differences were observed in species/lineage prevalence between the Western and Eastern regions. In Eastern Canada, 12.7% (*n* = 46) of the 362 section *Alternaria* strains were from the arborescens lineage, sampled from 14 different host genera. In Western Canada, only 2.2% (*n* = 1) of the 46 strains were arborescens, and that one strain was collected from an indoor biome (built environment). This difference is statistically significant (chi-square, *p* < 0.038), suggesting the distribution of the arborescens lineage in Canada is heavily biased towards the Eastern regions.

### Population genetic analyses: host/substrate source

Trait states for host/substrate isolation source were also mapped onto the haplotype network (Figure 5B). The 64 host genera were grouped into higher level taxonomic ranks to observe overall trends, but the main grass host genera were kept separate to explore potential associations. Similar to geography, there was a general lack of host partitioning of haplotypes of the more common species/lineages. For example, the alternata lineage was associated with all eight of the host sources, and the three most frequently recovered haplotypes were each sampled from seven host sources. The arborescens lineage was found on most hosts, with some smaller sub-clades showing potential host affinities (e.g., sub-clade 2 not sampled from monocots). No clear differences were observed between haplotypes sampled from wild plants versus agricultural crops. Strains collected from non-plant substrates, such as indoor biomes and soil, did not form distinct clusters in the haplotype network either.

### Recombination

Next, we searched for molecular signatures of within-lineage recombination in the sequence alignments for the alternata and arborescens lineages. No significant evidence of intra-locus recombination was found within any loci in either lineage. When the three loci were concatenated and analyzed together, some evidence for inter-locus recombination was observed: three and two recombination events were detected in the alternata and arborescens datasets, respectively (Supplementary Table S4). The peaks in probability distributions of breakpoints overlapped with the boundaries between loci, for both datasets, consistent with recombination between physically unlinked loci. The highly reticulate nature of the haplotype network for *A. alternata* (Figure 5) is suggestive of recombination as well. Finally, the mating-type idiomorph was determined for 166 of our newly characterized *A. alternata* strains (Supplementary Table S5). The ratio of MAT1-1 versus MAT1-2 was 72:94, which was not significantly different from the 50% split expected for a recombining population (chi-square, *p* > 0.22).

## Discussion

The central focus of this study was the species diversity and distribution of plant-associated *Alternaria* section *Alternaria* strains collected from within Canada. While 408 (73.1%) of the 558 analyzed strains originated from this country, strains from other countries were included to place Canadian diversity within the context of international diversity. In our large sample of strains from Canada, we recovered only two described section *Alternaria* species, with *A. alternata* (*n* = 360) being more common than *A. arborescens* (*n* = 47). One strain (KAS6097) represents an undescribed species that will be characterized in a future taxonomic study. Given that our Canadian strains were sampled from 46 different plant genera, and multiple indoor biomes, it may at first seem surprising that they all fell within only two described species. However, inspection of the descriptions of the numerous other known species within section *Alternaria* reveals that most of them are reported from tropical regions only, or from hosts that are not found in the temperate regions of North America (see Simmons, 2007; Woudenberg et al., 2015a). Even the absence from Canada of the moderately common lineages, such as gaisen and longipes, can be explained; the hosts typically associated with the gaisen (Asian pear) and longipes (tobacco, tangerines) lineages are not conventionally grown in Canada and therefore were not sampled.

Our report is the first to examine the diversity of section *Alternaria* strains from Canada using molecular markers that were specifically designed to distinguish between the closely related and morphologically similar species within this section. Our results are similar to previous reports from various other countries; many of these studies also found a high prevalence of *A. alternata* (or synonyms such as *A. tenuissima*), indicating this pattern may be quite general (Woudenberg et al., 2015b; Gao et al., 2021; Habib et al., 2021; Nichea et al., 2022). If we restrict our analyses to just Canadian strains from our top three cereal crops (*Triticum*, *Avena*, and *Hordeum*), 89.9% of these 317 strains were identified to *A. alternata*. If we narrow the focus to just Canadian strains from *Triticum* (*n* = 167), similar values are obtained (89.2% *A. alternata*). For direct comparison, recent studies of wheat-associated *Alternaria* have found *A. alternata* frequencies of 50–67% (Ramires et al., 2018; Somma et al., 2019; Masiello et al., 2020). Here, we found significant differences in species/lineage prevalence between Western and Eastern Canada, with *A. arborescens* being much more common in the Eastern regions. This finding is consistent with recent studies of *Alternaria* on wheat and other hosts (Kelman et al., 2020; Hafez et al., 2022), in which *A. arborescens* was not reported from any samples collected in Western Canada. Given that strains of *A. arborescens* may produce mycotoxin profiles that are different from *A. alternata* (Andersen et al., 2002, 2015; da Cruz Cabral et al., 2017; Kelman et al., 2020), information on the distribution of species, both by geography and host, is essential for predictive toxicological risk assessment.

Overall, our results are aligned with *A. alternata* being a globally widespread, genetically diverse species with little evidence for differentiation by geography or host association. Although some sub-clades appear in the combined tree (Figure 3), they were typically poorly supported and their separation was driven mainly by a single locus (e.g., alternata sub-clade 2 possessing a unique *ASA-19* allele). Characterization of additional loci, or full genome sequencing, will help to determine how consistently this sub-clade is recovered. Interestingly, all alternata sub-clade 2 strains were sampled from North America, but the strains were collected from eight different genera and the indoor biome. None of these strains were analyzed by us previously, or included in our marker development work.

The presence of multiple, well-supported sub-clades within the arborescens lineage (Figure 3) is consistent with the existence of multiple independent species. Previous work has referred to this group as the “*A. arborescens* species complex” (Woudenberg et al., 2015a; Dettman and Eggertson, 2021) which we use interchangeably with the “arborescens lineage” or “*A. arborescens*.” The seven strains with genomes reported by other authors have a limited breadth of diversity and are all restricted to arborescens sub-clade 2. Sub-clade 1, the largest, and sub-clade 3 are composed entirely of strains characterized by our research group. Thus, our sampling of new section *Alternaria* strains, mainly from Canada, has discovered novel sub-lineages and has significantly increased the known biodiversity of the main arborescens lineage. Here, the genetic diversity observed within the arborescens lineage was actually greater than that for the alternata lineage, despite the latter having a much greater sample size. At this point, we are not proposing any formal taxonomic or nomenclatural changes. While we do conclude that the arborescens lineage represents multiple species, further work is needed to determine if each sub-clade represents a single or multiple species. The inclusion of more internationally collected strains, and ex-type strains from described species in this complex, would also help to resolve this question. In contrast to alternata, the arborescens lineage displayed some evidence for uneven distribution by geography, but more comprehensive sampling is needed to explore further the host association patterns and potential sub-clade specificity.

To assess species diversity from large-scale plant pathology or biodiversity surveys, we require accurate and reliable tools with sufficient discriminatory power. The commonly employed internal transcribed spacer (ITS) of the rDNA region does not fulfill this requirement for very closely related taxa, such as those in *Alternaria* section *Alternaria*. Therefore, it is important to acknowledge that ITS-based metagenomic studies do not have the ability to resolve section *Alternaria* taxonomy down to the species level, and all species may be lumped into a single operational taxonomic unit (likely identified as “*A. alternata*”). Recognizing that the discriminatory power of standard molecular markers may be limited (Andrew et al., 2009; Armitage et al., 2015, 2020; Woudenberg et al., 2015a), we identified molecular markers whose evolutionary histories were congruent with phylogenomic relationships (Dettman and Eggertson, 2021) and demonstrated that they were informative and specific enough for routine diagnostics of the main lineages of section *Alternaria* (Dettman and Eggertson, 2022). Here, we further tested if the reliability of these diagnostic markers breaks down as they are challenged by larger strain samples that may contain previously undiscovered diversity. Overall, the *ASA-10* marker performed the best in terms of diagnostic ability (Figure 2, Supplementary Figure S1). The *ASA-19* locus was reliable for most lineages, however, the alternata lineage was divided into three clades (Figure 2, Supplementary Figure S2). Remarkably, the *ASA-10* and *ASA-19* markers had no direct conflicts between lineage assignment for any of the 558 strains. Given the non-monophyly of alternata in *ASA-19*, the *ASA-10* marker would be our clear choice for future single-locus diagnostic applications.

We characterized the *rpb2* locus for comparison to a reference gene that is already well represented in fungal molecular systematics datasets. Despite being one of the most informative standard housekeeping genes for systematics of section *Alternaria* (Woudenberg et al., 2015a; Dettman and Eggertson, 2021), here we found that *rpb2* had limited ability to distinguish lineages and its reliability continues to diminish as more strains are tested. Based on the results from the *ASA-10* and *ASA-19* markers, the *rpb2* locus placed five strains in the “incorrect” lineage (alternata versus arborescens). Given that *ASA* markers were chosen due to their congruence with phylogenomic relationships, we are inferring that *rpb2* is the anomalous locus. Regardless, the clear conflict between *rpb2* and *ASA* markers suggests that rare events of hybridization and recombination are occurring between lineages, resulting in alternata-arborescens hybrids. All five of these putative hybrid strains were isolated from wheat in regions of Ontario where alternata and arborescens lineages co-occur, demonstrating that the requirement of physical proximity for hybridization is met. Interestingly, two of the five putative hybrids (DET2289 and DET2290) were collected from the same field site. We acknowledge that conflict between locus genealogies could also be caused by incomplete sorting of ancestral polymorphism prior to lineage divergence, as explored previously for section *Alternaria* (Dettman and Eggertson, 2021). This process typically obscures the resolution of the phylogenetic relationships of the diverged taxa, and shared alleles tend to accrue mutations through time after the divergence event. However, the fact that the *rpb2* alleles in the putative hybrids are identical in sequence to an allele in the alternative lineage, for four of the five putative hybrids (Supplementary Figure S3), indicates that recent hybridization/recombination is the more plausible explanation. In our previous reports, we also found molecular evidence for potential hybrids between alternata and gaisen lineages, raising the questions: How common is inter-lineage hybridization? How does it affect the evolution and genomic composition of the species? More detailed analyses of these putative hybrids are required to answer these questions.

No sexual state has been documented, either in nature or the laboratory, for any species in section *Alternaria*. Similar to other putatively asexual fungi, investigations using a molecular genetic approach have consistently suggested that recombination does in fact occur (Andrew et al., 2009; Stewart et al., 2013, 2014; Meng et al., 2015; Adhikari et al., 2021, Dettman and Eggertson, 2021). In addition to the rare *between*-lineage hybridization mentioned above, further detailed investigation of our multi-locus molecular datasets revealed signatures of recombination *within* lineages. The three analyzed loci were located on different chromosomes (*ASA-10* on chr. 04; *ASA-19* on chr. 10; *rpb2* on chr. 05) and were therefore genetically unlinked. Evidence for between-locus recombination was observed within the alternata and arborescens lineages. The aforementioned putative hybrids were not contributing to the signatures of within-lineage recombination: none of the between-lineage hybrids were identified as within-lineage recombinants (Supplementary Table S4). Furthermore, in *A. alternata*, we found near equal frequencies of the two mating-type idiomorphs (MAT1-1 and MAT1-2) that interact to facilitate sexual reproduction in heterothallic Ascomycetes. This pattern suggests that the population undergoes sexual recombination often enough to allow frequency dependent selection to maintain a balanced distribution of the two idiomorphs. On a finer scale, no evidence of within-locus recombination was observed. Our ability to detect within-locus recombination may be limited because these diagnostic markers can have very little molecular variation within lineages (eg. *ASA-10* in alternata lineage) and recombination between identical alleles is not detectable. Whole-genome polymorphism analyses, combined with population-level sampling, is needed to reveal the full patterns of recombination in these taxa. Understanding the reproductive mode, and potential for adaptive evolution, of fungal pathogens is critical for disease prediction and management.

Although our sample size for section *Alternaria* is quite large, our sampling methodology was not systematic or standardized, so conclusions regarding species frequencies from particular sites or hosts may be affected by an unknown sampling bias. Strain isolation performed in our laboratory was blind and did not preferentially retain certain species over others. However, sampling bias may be greater for the strains with genomes that were sequenced and released into the public domain. Regardless, we believe our sampling of Canadian strains was comprehensive enough to reveal general patterns of species frequencies and distributions, at least for cereal crops. Here we report only on section *Alternaria*, so it should be noted that isolation attempts from various Canadian substrates/hosts have recovered strains from other sections, including sections *Infectoriae*, *Pseudoalternaria*, *Ulocladium*, *Pseudoulocladium*, *Ulocladioides*, and *Embellisia*. Of these sections, the most prevalent appears to be section *Infectoriae*, which is phylogenetically divergent from section *Alternaria* but similar in terms of taxonomically important characters such as asexual spore morphology. Other surveys of *Alternaria* diversity from cereal crops (Ramires et al., 2018; Somma et al., 2019; Hafez et al., 2022) have reported that section *Infectoriae* is common, and the unique mycotoxin profile of this section has been well established (Andersen et al., 2002; Patriarca et al., 2019). Further characterization of section *Infectoriae* strains sampled from Canadian crops is ongoing.

In conclusion, our analyses of the largest multi-locus sequence dataset for *Alternaria* section *Alternaria* to date provide new insights into species diversity and distributions. First, despite having at least 12 described species in the section, only *A. alternata* and *A. arborescens* were found in Canada, with the former being the most common. Significant differences in species prevalence were observed between Western and Eastern Canada, with *A. arborescens* being very rare in the Western regions. Second, our results indicate that *A. alternata* is a single, genetically diverse, globally distributed species with little differentiation by host or geography. In contrast, the arborescens lineage appears to be a species complex with multiple distinct sub-lineages and our sampling of new Canadian strains has greatly increased its breadth of characterized biodiversity. Third, rare events of recent inter-lineage hybridization may be occurring, producing apparent hybrid strains with mixed ancestry and genomic composition. Furthermore, observed molecular signatures of recombination suggest that cryptic sexual reproduction is occurring within these putatively asexual fungal species.

## Data availability statement

The datasets presented in this study can be found in online repositories. The names of the repository/repositories and accession number(s) can be found below: https://doi.org/10.5281/zenodo.7764657, Zenodo dataset.

## Author contributions

JD designed the project, obtained funding for the project, isolated and handled the fungal strains. QE and NK extracted DNA and performed molecular laboratory work. JD performed the data analyses. JD wrote the manuscript and QE and NK edited the manuscript. All authors contributed to the article and approved the submitted version.

## Funding

This study was supported by Agriculture and Agri-Food Canada under Grant J-002272.

## Conflict of interest

The authors declare that the research was conducted in the absence of any commercial or financial relationships that could be construed as a potential conflict of interest.

## Publisher’s note

All claims expressed in this article are solely those of the authors and do not necessarily represent those of their affiliated organizations, or those of the publisher, the editors and the reviewers. Any product that may be evaluated in this article, or claim that may be made by its manufacturer, is not guaranteed or endorsed by the publisher.
