# Supplementary material for: Species diversity and molecular characterization of Alternaria section Alternaria isolates collected mainly from cereal crops in Canada
Source: Front Microbiol. 2023 May 25;14:1194911. doi: 10.3389/fmicb.2023.1194911 (PMC10249498; doi:10.3389/fmicb.2023.1194911)
Supplement: Supplementary file 1 [file Data_Sheet_1.PDF]

Supplementary Material

Species diversity and molecular characterization of *Alternaria* section *Alternaria* isolates collected mainly from cereal crops in Canada

Jeremy R. Dettman*, Quinn Eggertson, and Natalie E. Kim

Agriculture and Agri-Food Canada, Ottawa Research and Development Centre, 960 Carling Avenue, Ottawa, Ontario, K1A 0C6, Canada

* Corresponding author: jeremy.dettman@agr.gc.ca

Supplementary Figure 1: Maximum likelihood tree constructed from the *ASA-10* locus. The 559 taxa labels are color-coded to indicate placement within phylogenetic lineages, as determined by combined analyses of the three loci together. Bootstrap percentages are shown on major branches. Taxon labels followed by "_genome" indicate sequences that were extracted from genome assemblies.

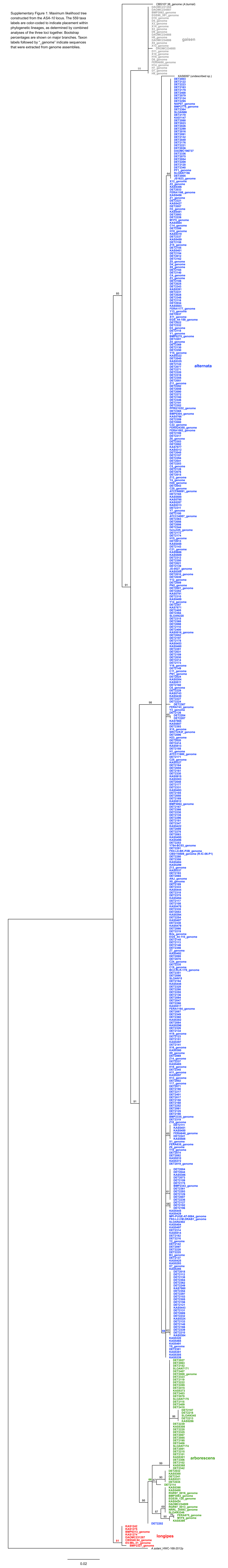

Supplementary Figure 2: Maximum likelihood tree constructed from the *ASA-19* locus. The 559 taxa labels are color-coded to indicate placement within phylogenetic lineages, as determined by combined analyses of the three loci together. Bootstrap percentages are shown on major branches. Taxon labels followed by "_genome" indicate sequences that were extracted from genome assemblies.

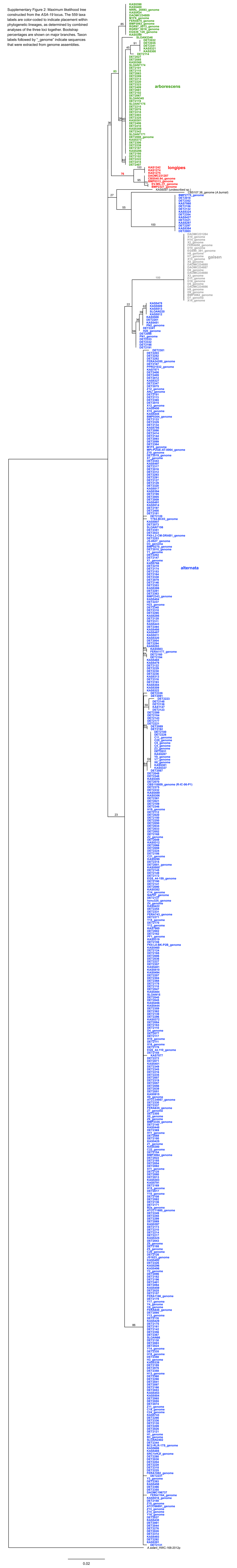

Supplementary Figure 3: Maximum likelihood tree constructed from the *rpb2* locus. The 559 taxa labels are color-coded to indicate placement within phylogenetic lineages, as determined by combined analyses of the three loci together. Bootstrap percentages are shown on major branches. Taxon labels followed by "_genome" indicate sequences that were extracted from genome assemblies.

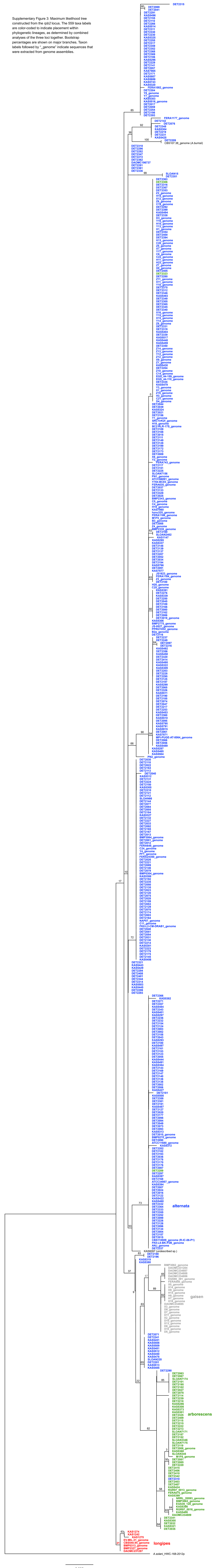

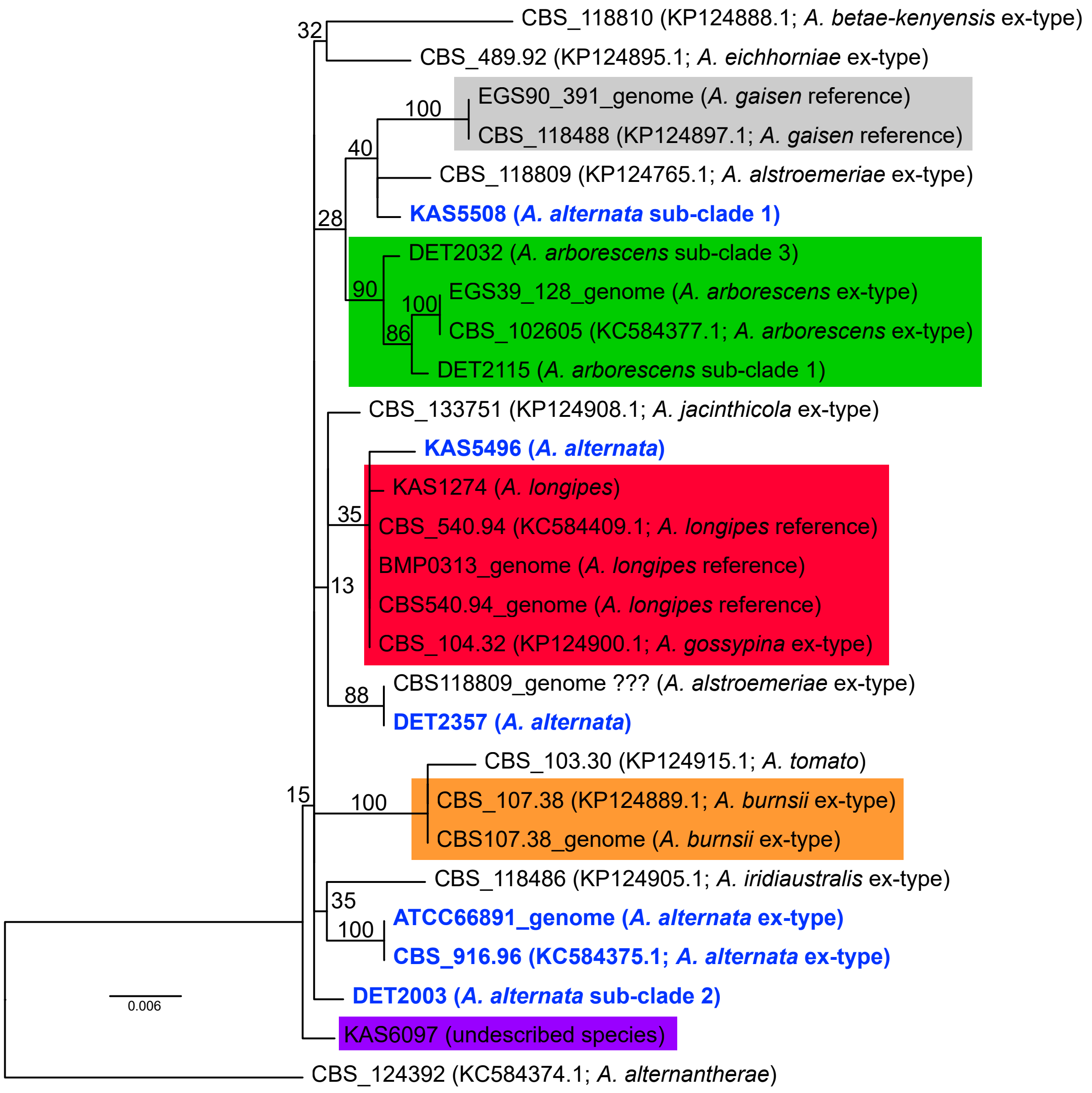

Supplementary Figure 4: Maximum likelihood tree constructed from representative and reference sequences for the *rpb2* locus. Bootstrap percentages are shown on major branches. NCBI accession numbers are listed in taxon labels, if applicable.

Sequences generated from different samples of the same strain, sometimes with synonymous identifiers, were included to assess internal consistency of public data:
A. gaisen reference: EGS90_391 genome versus CBS_118488 Sanger.
A. arborescens ex-type: EGS39_128 genome versus CBS_102605 Sanger.
A. longipes reference: CBS540.94 genome versus BMP0313 genome versus CBS_540.94 Sanger.
A. burnsii ex-type: CBS107.38 genome versus CBS_107.38 Sanger.
A. alternata ex-type: ATCC66891 genome versus CBS_916.96 Sanger.
A. alstroemeriae ex-type: CBS118809 genome versus CBS_118809 Sanger.

The only case where sequences did not match as expected was the *A. alstroemeriae* ex-type strain (CBS_118809). The Sanger sequence for this strain was unique and closely related to *A. gaisen*, consistent with previous multi-locus phylogenetic analyses (Woudenberg et al. 2015). The sequence extracted from the genome assembly, however, was indistinguishable from *A. alternata*. Sequences of the *ASA-10* and *ASA-19* loci extracted from the genome assembly were also indistinguishable from *A. alternata* (Supplementary Figures 1 and 2). Investigation into available metadata and submission information revealed that details of the genome assembly strain (eg. date, location, and isolation source) did not match that for the ex-type of *A. alstroemeriae*. Therefore, we concluded that the Sanger sequence accession is correct, and the genome assembly is likely an *A. alternata* strain mis-identified as *A. alstroemeriae*. For all analyses, this genome was considered to represent an *A. alternata* strain. The only exception was when this strain was not included in recombination analyses of the *A. alternata* dataset.

Supplementary Table 1: Information on the 385 newly characterized *Alternaria* section *Alternaria* strains.

Isolate Name	Species	Substrate Source	Collection Year	Country	Province/State	City/Region
DET2002	*A. alternata*	*Triticum*	2018	Canada	Ontario	Grand Valley
DET2003	*A. alternata*	*Triticum*	2018	Canada	Ontario	Grand Valley
DET2004	*A. alternata*	*Triticum*	2018	Canada	Ontario	Clearview
DET2005	*A. alternata*	*Triticum*	2018	Canada	Ontario	Clearview
DET2006	*A. alternata*	*Triticum*	2018	Canada	Ontario	Clearview
DET2007	*A. alternata*	*Triticum*	2018	Canada	Ontario	Clearview
DET2009	*A. alternata*	*Triticum*	2018	Canada	Ontario	Clearview
DET2012	*A. alternata*	*Triticum*	2018	Canada	Ontario	Southgate
DET2013	*A. alternata*	*Triticum*	2018	Canada	Ontario	Southgate
DET2016	*A. alternata*	*Triticum*	2018	Canada	Ontario	Conn (Southgate)
DET2017	*A. alternata*	*Triticum*	2018	Canada	Ontario	Conn (Southgate)
DET2018	*A. alternata*	*Triticum*	2018	Canada	Ontario	Atwood
DET2020	*A. alternata*	*Triticum*	2018	Canada	Ontario	Perth East
DET2021	*A. alternata*	*Triticum*	2018	Canada	Ontario	Perth East
DET2022	*A. alternata*	*Triticum*	2018	Canada	Ontario	Perth East
DET2023	*A. alternata*	*Triticum*	2018	Canada	Ontario	Stouffville (Uxbridge)
DET2024	*A. alternata*	*Triticum*	2018	Canada	Ontario	Stouffville (Uxbridge)
DET2025	*A. alternata*	*Triticum*	2018	Canada	Ontario	South Dundas
DET2026	*A. alternata*	*Triticum*	2018	Canada	Ontario	South Dundas
DET2027	*A. arborescens*	*Triticum*	2018	Canada	Ontario	South Dundas
DET2028	*A. alternata*	*Triticum*	2018	Canada	Ontario	Grand Valley
DET2030	*A. alternata*	*Triticum*	2018	Canada	Ontario	Grand Valley
DET2031	*A. alternata*	*Triticum*	2018	Canada	Ontario	Clearview
DET2032	*A. arborescens*	*Triticum*	2018	Canada	Ontario	Clearview
DET2033	*A. alternata*	*Triticum*	2018	Canada	Ontario	Winchester
DET2034	*A. alternata*	*Triticum*	2018	Canada	Ontario	Russell
DET2035	*A. arborescens*	*Triticum*	2018	Canada	Ontario	Clearview
DET2036	*A. alternata*	*Triticum*	2018	Canada	Ontario	Clearview
DET2037	*A. alternata*	*Triticum*	2018	Canada	Ontario	Nottawasaga
DET2039	*A. alternata*	*Triticum*	2018	Canada	Ontario	Russell
DET2040	*A. alternata*	*Triticum*	2018	Canada	Ontario	Singhampton (Clearview)
DET2041	*A. alternata*	*Triticum*	2018	Canada	Ontario	Singhampton (Clearview)
DET2043	*A. alternata*	*Triticum*	2018	Canada	Ontario	Southgate
DET2044	*A. alternata*	*Triticum*	2018	Canada	Ontario	Southgate
DET2045	*A. alternata*	*Triticum*	2018	Canada	Ontario	Southgate
DET2047	*A. alternata*	*Triticum*	2018	Canada	Ontario	Russell
DET2048	*A. alternata*	*Triticum*	2018	Canada	Ontario	St. Isidore
DET2049	*A. alternata*	*Triticum*	2018	Canada	Ontario	Harriston
DET2050	*A. alternata*	*Triticum*	2018	Canada	Ontario	Harriston
DET2051	*A. alternata*	*Triticum*	2018	Canada	Ontario	Harriston
DET2052	*A. alternata*	*Triticum*	2018	Canada	Ontario	Atwood
DET2053	*A. alternata*	*Triticum*	2018	Canada	Ontario	Atwood
DET2054	*A. alternata*	*Triticum*	2018	Canada	Ontario	Atwood
DET2055	*A. alternata*	*Triticum*	2018	Canada	Ontario	St.Isidore
DET2056	*A. alternata*	*Triticum*	2018	Canada	Ontario	St.Isidore
DET2057	*A. alternata*	*Triticum*	2018	Canada	Ontario	Alfred and Plantagenet
DET2059	*A. alternata*	*Triticum*	2018	Canada	Ontario	Alfred and Plantagenet
DET2061	*A. alternata*	*Triticum*	2018	Canada	Ontario	Alfred and Plantagenet
DET2063	*A. arborescens*	*Triticum*	2018	Canada	Ontario	Alfred and Plantagenet
DET2065	*A. alternata*	*Triticum*	2018	Canada	Ontario	Champlain
DET2066	*A. alternata*	*Triticum*	2018	Canada	Ontario	Champlain
DET2067	*A. arborescens*	*Triticum*	2018	Canada	Ontario	North Dundas
DET2069	*A. alternata*	*Triticum*	2018	Canada	Ontario	Perth East
DET2070	*A. alternata*	*Triticum*	2018	Canada	Ontario	Perth East
DET2073	*A. alternata*	*Triticum*	2018	Canada	Ontario	South Dundas
DET2074	*A. alternata*	*Triticum*	2018	Canada	Ontario	South Dundas
DET2075	*A. alternata*	*Triticum*	2018	Canada	Ontario	Winchester
DET2077	*A. alternata*	*Triticum*	2018	Canada	Ontario	Winchester
DET2079	*A. alternata*	*Triticum*	2018	Canada	Ontario	Russell
DET2081	*A. arborescens*	*Triticum*	2018	Canada	Ontario	Russell
DET2082	*A. alternata*	*Triticum*	2018	Canada	Ontario	Russell
DET2083	*A. alternata*	*Triticum*	2018	Canada	Ontario	St.Isidore

DET2084	*A. alternata*	*Triticum*	2018	Canada	Ontario	St.Isidore
DET2086	*A. alternata*	*Triticum*	2018	Canada	Ontario	St.Isidore
DET2087	*A. alternata*	*Triticum*	2018	Canada	Ontario	St.Isidore
DET2088	*A. alternata*	*Triticum*	2018	Canada	Ontario	Alfred and Plantagenet
DET2089	*A. alternata*	*Triticum*	2018	Canada	Ontario	Alfred and Plantagenet
DET2090	*A. alternata*	*Triticum*	2018	Canada	Ontario	Alfred and Plantagenet
DET2091	*A. alternata*	*Triticum*	2018	Canada	Ontario	Alfred and Plantagenet
DET2092	*A. alternata*	*Triticum*	2018	Canada	Ontario	Alfred and Plantagenet
DET2093	*A. alternata*	*Triticum*	2018	Canada	Ontario	Alfred and Plantagenet
DET2094	*A. alternata*	*Triticum*	2018	Canada	Ontario	Alfred and Plantagenet
DET2095	*A. alternata*	*Triticum*	2018	Canada	Ontario	Alfred and Plantagenet
DET2096	*A. alternata*	*Triticum*	2018	Canada	Ontario	Alfred and Plantagenet
DET2097	*A. alternata*	*Triticum*	2018	Canada	Ontario	Alfred and Plantagenet
DET2098	*A. alternata*	*Triticum*	2018	Canada	Ontario	Alfred and Plantagenet
DET2099	*A. alternata*	*Triticum*	2018	Canada	Ontario	Champlain
DET2100	*A. alternata*	*Triticum*	2018	Canada	Ontario	Champlain
DET2101	*A. arborescens*	*Triticum*	2018	Canada	Ontario	North Dundas
DET2102	*A. arborescens*	*Triticum*	2018	Canada	Ontario	North Dundas
DET2108	*A. alternata*	*Avena sativa*	2018	Canada	Saskatchewan	Melfort
DET2109	*A. alternata*	*Avena sativa*	2018	Canada	Saskatchewan	Melfort
DET2110	*A. alternata*	*Avena sativa*	2018	Canada	Saskatchewan	Melfort
DET2111	*A. alternata*	*Avena sativa*	2018	Canada	Saskatchewan	Melfort
DET2112	*A. alternata*	*Avena sativa*	2018	Canada	Quebec	Normandin
DET2113	*A. alternata*	*Avena sativa*	2018	Canada	Quebec	Normandin
DET2114	*A. arborescens*	*Avena sativa*	2018	Canada	Quebec	Normandin
DET2115	*A. arborescens*	*Avena sativa*	2018	Canada	Quebec	Normandin
DET2116	*A. alternata*	*Avena sativa*	2018	Canada	Ontario	New Liskeard
DET2117	*A. alternata*	*Avena sativa*	2018	Canada	Ontario	New Liskeard
DET2118	*A. alternata*	*Avena sativa*	2018	Canada	Ontario	New Liskeard
DET2119	*A. arborescens*	*Avena sativa*	2018	Canada	Ontario	Ottawa
DET2120	*A. alternata*	*Avena sativa*	2018	Canada	Ontario	Ottawa
DET2121	*A. alternata*	*Avena sativa*	2018	Canada	Ontario	Ottawa
DET2122	*A. alternata*	*Avena sativa*	2018	Canada	Ontario	Horning's Mills
DET2124	*A. alternata*	*Avena sativa*	2018	Canada	Ontario	Horning's Mills
DET2126	*A. alternata*	*Avena sativa*	2018	Canada	Ontario	Priceville
DET2127	*A. alternata*	*Avena sativa*	2018	Canada	Ontario	Priceville
DET2128	*A. alternata*	*Avena sativa*	2018	Canada	Ontario	Southgate
DET2129	*A. alternata*	*Avena sativa*	2018	Canada	Ontario	Southgate
DET2130	*A. alternata*	*Avena sativa*	2018	Canada	Ontario	Southgate
DET2131	*A. alternata*	*Avena sativa*	2018	Canada	Ontario	Kenilworth
DET2132	*A. alternata*	*Avena sativa*	2018	Canada	Ontario	Kenilworth
DET2133	*A. alternata*	*Avena sativa*	2018	Canada	Ontario	Kenilworth
DET2134	*A. alternata*	*Avena sativa*	2018	Canada	Ontario	Kenilworth
DET2135	*A. alternata*	*Avena sativa*	2018	Canada	Ontario	Mt. Forest
DET2136	*A. alternata*	*Avena sativa*	2018	Canada	Ontario	Mt. Forest
DET2137	*A. alternata*	*Avena sativa*	2018	Canada	Ontario	Mt. Forest
DET2138	*A. alternata*	*Avena sativa*	2018	Canada	Ontario	Mt. Forest
DET2139	*A. alternata*	*Avena sativa*	2018	Canada	Ontario	Mt. Forest
DET2140	*A. alternata*	*Avena sativa*	2018	Canada	Ontario	Mt. Forest
DET2141	*A. alternata*	*Avena sativa*	2018	Canada	Ontario	Warkworth
DET2142	*A. alternata*	*Avena sativa*	2018	Canada	Ontario	Warkworth
DET2143	*A. alternata*	*Avena sativa*	2018	Canada	Ontario	Warkworth
DET2144	*A. alternata*	*Hordeum vulgare*	2018	Canada	Ontario	Southgate?
DET2145	*A. alternata*	*Hordeum vulgare*	2018	Canada	Ontario	Southgate?
DET2146	*A. alternata*	*Hordeum vulgare*	2018	Canada	Ontario	Southgate?
DET2147	*A. alternata*	*Hordeum vulgare*	2018	Canada	Ontario	Southgate
DET2148	*A. alternata*	*Hordeum vulgare*	2018	Canada	Ontario	Southgate
DET2149	*A. alternata*	*Hordeum vulgare*	2018	Canada	Ontario	Southgate
DET2150	*A. alternata*	*Hordeum vulgare*	2018	Canada	Ontario	Dundalk
DET2151	*A. alternata*	*Hordeum vulgare*	2018	Canada	Ontario	Dundalk
DET2152	*A. arborescens*	*Hordeum vulgare*	2018	Canada	Ontario	Dundalk
DET2153	*A. alternata*	*Hordeum vulgare*	2018	Canada	Ontario	Southgate
DET2154	*A. alternata*	*Hordeum vulgare*	2018	Canada	Ontario	Southgate
DET2155	*A. alternata*	*Hordeum vulgare*	2018	Canada	Ontario	Southgate
DET2156	*A. alternata*	*Hordeum vulgare*	2018	Canada	Ontario	Conn (Southgate)

DET2157	*A. alternata*	*Hordeum vulgare*	2018	Canada	Ontario	Conn (Southgate)
DET2158	*A. alternata*	*Hordeum vulgare*	2018	Canada	Ontario	Conn (Southgate)
DET2159	*A. alternata*	*Hordeum vulgare*	2018	Canada	Ontario	Mt. Forest
DET2160	*A. alternata*	*Hordeum vulgare*	2018	Canada	Ontario	Mt. Forest
DET2161	*A. alternata*	*Hordeum vulgare*	2018	Canada	Ontario	Mt. Forest
DET2162	*A. alternata*	*Hordeum vulgare*	2018	Canada	Ontario	Mt. Forest
DET2163	*A. alternata*	*Hordeum vulgare*	2018	Canada	Ontario	Mt. Forest
DET2164	*A. alternata*	*Hordeum vulgare*	2018	Canada	Ontario	Mt. Forest
DET2165	*A. alternata*	*Hordeum vulgare*	2018	Canada	Ontario	Mt. Forest
DET2166	*A. alternata*	*Hordeum vulgare*	2018	Canada	Ontario	Mt. Forest
DET2167	*A. alternata*	*Hordeum vulgare*	2018	Canada	Ontario	Minto
DET2168	*A. alternata*	*Hordeum vulgare*	2018	Canada	Ontario	Minto
DET2169	*A. alternata*	*Hordeum vulgare*	2018	Canada	Ontario	Minto
DET2170	*A. alternata*	*Hordeum vulgare*	2018	Canada	Ontario	Palmerston (Minto)
DET2171	*A. alternata*	*Hordeum vulgare*	2018	Canada	Ontario	Palmerston (Minto)
DET2172	*A. alternata*	*Hordeum vulgare*	2018	Canada	Ontario	Palmerston (Minto)
DET2173	*A. alternata*	*Hordeum vulgare*	2018	Canada	Ontario	Huron East
DET2174	*A. alternata*	*Hordeum vulgare*	2018	Canada	Ontario	Huron East
DET2175	*A. alternata*	*Hordeum vulgare*	2018	Canada	Ontario	Huron East
DET2176	*A. alternata*	*Hordeum vulgare*	2018	Canada	Ontario	Trent Hills
DET2177	*A. alternata*	*Hordeum vulgare*	2018	Canada	Ontario	Trent Hills
DET2178	*A. alternata*	*Hordeum vulgare*	2018	Canada	Ontario	Trent Hills
DET2179	*A. alternata*	*Hordeum vulgare*	2018	Canada	Ontario	Casselman (The Nation)
DET2180	*A. alternata*	*Hordeum vulgare*	2018	Canada	Ontario	Casselman (The Nation)
DET2181	*A. alternata*	*Hordeum vulgare*	2018	Canada	Ontario	Casselman (The Nation)
DET2182	*A. alternata*	*Hordeum vulgare*	2018	Canada	Ontario	Casselman (The Nation)
DET2184	*A. alternata*	*Hordeum vulgare*	2018	Canada	Ontario	Casselman (The Nation)
DET2185	*A. alternata*	*Hordeum vulgare*	2018	Canada	Ontario	Vernon
DET2187	*A. arborescens*	*Hordeum vulgare*	2018	Canada	Ontario	Vernon
DET2189	*A. alternata*	*Poa*	2018	Canada	Quebec	Rimouski
DET2190	*A. alternata*	*Poa*	2018	Canada	Quebec	Alma
DET2191	*A. alternata*	*Poa*	2018	Canada	Quebec	Alma
DET2192	*A. alternata*	*Poa*	2018	Canada	Quebec	Alma
DET2193	*A. alternata*	*Calamagrostis*	2018	Canada	Quebec	Parc National Pointe-Taillon
DET2194	*A. alternata*	*Calamagrostis*	2018	Canada	Quebec	Parc National Pointe-Taillon
DET2195	*A. arborescens*	*Phalaris*	2018	Canada	Quebec	Parc National Pointe-Taillon
DET2197	*A. alternata*	*Poa/Agrostis*	2018	Canada	Quebec	north to La Tuque
DET2198	*A. alternata*	*Poa/Agrostis*	2018	Canada	Quebec	north to La Tuque
DET2199	*A. alternata*	*Cirsium undulatus*	1999	Canada	Alberta	
DET2202	*A. alternata*	*Lomatium triternatum*	1999	Canada	Alberta	
DET2203	*A. alternata*	*Thermopsis rhombifolia*	1999	Canada	Alberta	
DET2204	*A. alternata*	*Avena sativa*	2019	Canada	Ontario	Ottawa
DET2207	*A. alternata*	*Avena sativa*	2019	Canada	Ontario	Ottawa
DET2210	*A. alternata*	*Avena sativa*	2019	Canada	Ontario	Ottawa
DET2213	*A. arborescens*	*Avena sativa*	2019	Canada	Ontario	Ottawa
DET2214	*A. alternata*	*Avena sativa*	2019	Canada	Ontario	Ottawa
DET2215	*A. arborescens*	*Zea mays*	2019	Canada	Ontario	Ottawa
DET2216	*A. alternata*	*Glycine max*	2019	Canada	Ontario	Ottawa
DET2217	*A. alternata*	*Glycine max*	2019	Canada	Ontario	Ottawa
DET2218	*A. arborescens*	*Glycine max*	2019	Canada	Ontario	Ottawa
DET2220	*A. arborescens*	*Triticum*	2018	Canada	Quebec	Rigaud
DET2221	*A. alternata*	*Triticum*	2018	Canada	Quebec	Rigaud
DET2222	*A. arborescens*	*Triticum*	2018	Canada	Quebec	Rigaud
DET2223	*A. alternata*	*Triticum*	2018	Canada	Quebec	
DET2224	*A. alternata*	*Triticum*	2018	Canada	Quebec	
DET2225	*A. alternata*	*Triticum*	2018	Canada	Quebec	
DET2226	*A. alternata*	*Triticum*	2018	Canada	Quebec	
DET2227	*A. alternata*	*Triticum*	2018	Canada	Quebec	
DET2228	*A. alternata*	*Triticum*	2018	Canada	Quebec	
DET2229	*A. alternata*	*Triticum*	2018	Canada	Quebec	
DET2230	*A. alternata*	*Triticum*	2018	Canada	Quebec	
DET2232	*A. alternata*	*Triticum*	2018	Canada	Quebec	
DET2235	*A. alternata*	*Triticum*	2018	Canada	Quebec	
DET2236	*A. alternata*	*Triticum*	2018	Canada	Quebec	
DET2237	*A. alternata*	*Triticum*	2018	Canada	Quebec	

DET2239	*A. arborescens*	*Triticum*	2018	Canada	Quebec	
DET2249	*A. alternata*	*Triticum*	2018	Canada	Quebec	
DET2250	*A. alternata*	*Triticum*	2018	Canada	Quebec	
DET2252	*A. alternata*	*Triticum*	2018	Canada	Quebec	
DET2253	*A. alternata*	*Triticum*	2018	Canada	Quebec	
DET2254	*A. alternata*	*Triticum*	2018	Canada	Quebec	
DET2255	*A. alternata*	*Triticum*	2018	Canada	Quebec	
DET2266	*A. alternata*	*Triticum*	2019	Canada	Ontario	Amaranth
DET2271	*A. alternata*	*Triticum*	2019	Canada	Ontario	Amaranth
DET2276	*A. alternata*	*Triticum*	2019	Canada	Ontario	East Garafraxa
DET2280	*A. alternata*	*Triticum*	2019	Canada	Ontario	Mapleton
DET2281	*A. alternata*	*Triticum*	2019	Canada	Ontario	Mapleton
DET2282	*A. alternata*	*Triticum*	2019	Canada	Ontario	Arthur
DET2289	*A. arborescens*	*Triticum*	2019	Canada	Ontario	Mt Forest
DET2290	*A. alternata*	*Triticum*	2019	Canada	Ontario	Mt Forest
DET2291	*A. alternata*	*Triticum*	2019	Canada	Ontario	Mt Forest
DET2292	*A. alternata*	*Triticum*	2019	Canada	Ontario	Mt Forest
DET2293	*A. alternata*	*Triticum*	2019	Canada	Ontario	Mt Forest
DET2294	*A. alternata*	*Triticum*	2019	Canada	Ontario	Mt Forest
DET2295	*A. alternata*	*Triticum*	2019	Canada	Ontario	Harriston
DET2296	*A. alternata*	*Triticum*	2019	Canada	Ontario	Harriston
DET2297	*A. alternata*	*Triticum*	2019	Canada	Ontario	Listowel (North Perth)
DET2304	*A. alternata*	*Triticum*	2019	Canada	Ontario	Erin Tlin
DET2305	*A. alternata*	*Triticum*	2019	Canada	Ontario	Erin Tlin
DET2306	*A. arborescens*	*Triticum*	2019	Canada	Ontario	Erin Tlin
DET2310	*A. alternata*	*Triticum*	2019	Canada	Ontario	Port Perry
DET2311	*A. alternata*	*Triticum*	2019	Canada	Ontario	Port Perry
DET2312	*A. alternata*	*Triticum*	2019	Canada	Ontario	Winchester
DET2314	*A. alternata*	*Triticum*	2019	Canada	Ontario	Winchester
DET2315	*A. alternata*	*Triticum*	2019	Canada	Ontario	Winchester
DET2316	*A. alternata*	*Triticum*	2019	Canada	Ontario	Edwards
DET2317	*A. alternata*	*Triticum*	2019	Canada	Ontario	Edwards
DET2318	*A. alternata*	*Triticum*	2019	Canada	Ontario	Russell
DET2319	*A. alternata*	*Triticum*	2019	Canada	Ontario	Russell
DET2320	*A. alternata*	*Triticum*	2019	Canada	Ontario	Casselman
DET2321	*A. alternata*	*Triticum*	2019	Canada	Ontario	Casselman
DET2323	*A. arborescens*	*Triticum*	2019	Canada	Ontario	St Isidore
DET2325	*A. arborescens*	*Triticum*	2019	Canada	Ontario	St Isidore
DET2326	*A. alternata*	*Triticum*	2019	Canada	Ontario	St Isidore
DET2329	*A. alternata*	*Triticum*	2019	Canada	Ontario	St Isidore
DET2330	*A. alternata*	*Triticum*	2019	Canada	Ontario	St Isidore
DET2331	*A. alternata*	*Solanum*	2019	Canada	Ontario	Ottawa
DET2332	*A. alternata*	*Hosta*	2019	Canada	Ontario	Ottawa
DET2333	*A. alternata*	*Hosta*	2019	Canada	Ontario	Ottawa
DET2335	*A. alternata*	*Avena sativa*	2019	Canada	Ontario	Kemptville
DET2336	*A. alternata*	*Avena sativa*	2019	Canada	Ontario	Caledon
DET2337	*A. alternata*	*Avena sativa*	2019	Canada	Ontario	Caledon
DET2338	*A. alternata*	*Avena sativa*	2019	Canada	Ontario	Centre Wellington
DET2339	*A. alternata*	*Avena sativa*	2019	Canada	Ontario	Centre Wellington
DET2340	*A. alternata*	*Avena sativa*	2019	Canada	Ontario	Minto
DET2341	*A. arborescens*	*Avena sativa*	2019	Canada	Ontario	Minto
DET2342	*A. arborescens*	*Avena sativa*	2019	Canada	Ontario	Clifford (Minto)
DET2343	*A. alternata*	*Avena sativa*	2019	Canada	Ontario	Clifford (Minto)
DET2344	*A. alternata*	*Avena sativa*	2019	Canada	Ontario	Harriston
DET2345	*A. alternata*	*Avena sativa*	2019	Canada	Ontario	Harriston
DET2346	*A. alternata*	*Avena sativa*	2019	Canada	Ontario	Gowanstown
DET2347	*A. alternata*	*Avena sativa*	2019	Canada	Ontario	Gowanstown
DET2348	*A. alternata*	*Avena sativa*	2019	Canada	Ontario	North Perth
DET2349	*A. alternata*	*Avena sativa*	2019	Canada	Ontario	North Perth
DET2350	*A. alternata*	*Avena sativa*	2019	Canada	Ontario	Perth East
DET2352	*A. alternata*	*Avena sativa*	2019	Canada	Ontario	Milverton
DET2353	*A. alternata*	*Avena sativa*	2019	Canada	Ontario	Milverton
DET2354	*A. alternata*	*Avena sativa*	2019	Canada	Ontario	Milverton
DET2355	*A. alternata*	*Avena sativa*	2019	Canada	Ontario	Milverton
DET2356	*A. alternata*	*Avena sativa*	2019	Canada	Ontario	Milverton

DET2357	*A. alternata*	*Avena sativa*	2019	Canada	Ontario	Milverton
DET2358	*A. alternata*	*Avena sativa*	2019	Canada	Ontario	East Garafraxa
DET2359	*A. alternata*	*Avena sativa*	2019	Canada	Ontario	East Garafraxa
DET2360	*A. alternata*	*Avena sativa*	2019	Canada	Ontario	Schomberg
DET2361	*A. alternata*	*Avena sativa*	2019	Canada	Ontario	Schomberg
DET2362	*A. alternata*	*Avena sativa*	2019	Canada	Ontario	Port Perry
DET2363	*A. alternata*	*Avena sativa*	2019	Canada	Ontario	Port Perry
DET2364	*A. alternata*	*Avena sativa*	2019	Canada	Ontario	Millbrook
DET2365	*A. alternata*	*Avena sativa*	2019	Canada	Ontario	Millbrook
DET2366	*A. alternata*	*Avena sativa*	2019	Canada	Ontario	Edwardsburgh/Cardinal
DET2367	*A. alternata*	*Avena sativa*	2019	Canada	Ontario	Edwardsburgh/Cardinal
DET2368	*A. alternata*	*Avena sativa*	2019	Canada	Ontario	South Mountain
DET2369	*A. alternata*	*Avena sativa*	2019	Canada	Ontario	Milverton
DET2375	*A. alternata*	*Hordeum vulgare*	2019	Canada	Ontario	Mt Forest
DET2381	*A. alternata*	*Hordeum vulgare*	2019	Canada	Ontario	Clifford (Minto)
DET2393	*A. alternata*	*Hordeum vulgare*	2019	Canada	Ontario	Schomberg (King)
DET2394	*A. alternata*	*Hordeum vulgare*	2019	Canada	Ontario	Schomberg
DET2399	*A. alternata*	*Hordeum vulgare*	2019	Canada	Ontario	Metcalfe
DET2400	*A. alternata*	*Hordeum vulgare*	2019	Canada	Ontario	Metcalfe
DET2401	*A. alternata*	*Hordeum vulgare*	2019	Canada	Ontario	Vars
DET2403	*A. arborescens*	*Hordeum vulgare*	2019	Canada	Ontario	St Isidore
DET2405	*A. alternata*	*Avena sativa*	2019	Canada	Quebec	Beauharnois
DET2406	*A. arborescens*	*Avena sativa*	2019	Canada	Quebec	Beauharnois
DET2407	*A. arborescens*	*Avena sativa*	2019	Canada	Quebec	Beauharnois
DET2408	*A. alternata*	*Avena sativa*	2019	Canada	Quebec	Beauharnois
DET2409	*A. arborescens*	*Avena sativa*	2019	Canada	Quebec	St-Clotilde-De-Horton
DET2410	*A. arborescens*	*Avena sativa*	2019	Canada	Quebec	St-Clotilde-De-Horton
DET2414	*A. alternata*	*Avena sativa*	2019	Canada	Quebec	St Pierre
DET2415	*A. arborescens*	*Avena sativa*	2019	Canada	Quebec	St Pierre
KAS1147	*A. alternata*	soil	1996	South Africa	KwaZulu-Natal	
KAS1242	*A. longipes*	*Astronium suaveolens*	1999	Costa Rica	San Jose	Puriscal
KAS5286	*A. arborescens*	*Rosa*	2014	Canada	Ontario	Ottawa-Carleton
KAS5287	*A. alternata*	*Hedera*	2014	Canada	Ontario	Ottawa-Carleton
KAS5293	*A. alternata*	*Pilosella aurantiaca*	2014	Canada	Ontario	Ottawa-Carleton
KAS5295	*A. alternata*	*Hippeastrum*	2014	Canada	Ontario	Ottawa-Carleton
KAS5296	*A. alternata*	*Salix*	2014	Canada	Ontario	Ottawa-Carleton
KAS5297	*A. alternata*	*Lewisia longipetala*	2014	Canada	Ontario	Ottawa-Carleton
KAS5299	*A. alternata*	*Lewisia longipetala*	2014	Canada	Ontario	Ottawa-Carleton
KAS5300	*A. arborescens*	*Tamarix ramosissima*	2014	Canada	Ontario	Ottawa-Carleton
KAS5301	*A. arborescens*	*Brassica*	2014	Canada	Ontario	Ottawa-Carleton
KAS5304	*A. alternata*	*Wiegela*	2014	Canada	Ontario	Ottawa-Carleton
KAS5305	*A. alternata*	*Juniperus virginiana*	2014	Canada	Ontario	Ottawa-Carleton
KAS5306	*A. alternata*	*Ipomea batatas*	2014	Canada	Ontario	Ottawa-Carleton
KAS5308	*A. arborescens*	*Malus baccata*	2014	Canada	Ontario	Ottawa-Carleton
KAS5309	*A. alternata*	*Aesculus flava*	2014	Canada	Ontario	Ottawa-Carleton
KAS5312	*A. alternata*	*Pinus*	2014	Canada	Ontario	Ottawa-Carleton
KAS5313	*A. alternata*	*Solanum lycopersicum*	2014	Canada	Ontario	Ottawa-Carleton
KAS5320	*A. alternata*	*Daucus carota*	2014	Canada	Ontario	Ottawa-Carleton
KAS5321	*A. arborescens*	*Solanum lycopersicum*	2014	Canada	Ontario	Ottawa-Carleton
KAS5322	*A. alternata*	*Acer*	2014	Canada	Ontario	Ottawa-Carleton
KAS5324	*A. alternata*	*Delphinium*	2014	Canada	Ontario	Ottawa-Carleton
KAS5325	*A. alternata*	*Scirpus*	2014	Canada	Ontario	Ottawa-Carleton
KAS5327	*A. alternata*	*Hibiscus rosa-sinensis*	2014	Canada	Ontario	Ottawa-Carleton
KAS5337	*A. alternata*	*Rhus typhina*	2014	Canada	Ontario	Ottawa-Carleton
KAS5339	*A. alternata*	*Dicentra canadensis*	2014	Canada	Ontario	Ottawa-Carleton
KAS5372	*A. alternata*	*Asclepias*	2014	Canada	Ontario	Ottawa-Carleton
KAS5373	*A. arborescens*	*Silene vulgaris*	2014	Canada	Ontario	Ottawa-Carleton
KAS5381	*A. alternata*	*Ipomea*	2014	Canada	Ontario	Ottawa-Carleton
KAS5382	*A. alternata*	*Ipomea*	2014	Canada	Ontario	Ottawa-Carleton
KAS5384	*A. alternata*	*Begonia*	2014	Canada	Ontario	Ottawa-Carleton
KAS5391	*A. alternata*	*Brunnera macrophylla*	2014	Canada	Ontario	Ottawa-Carleton
KAS5394	*A. alternata*	*Solanum lycopersicum*	2014	Canada	Ontario	Ottawa-Carleton
KAS5397	*A. alternata*	*Cucurbita pepo*	2014	Canada	Ontario	Goulbourn Twp.
KAS5398	*A. arborescens*	*Malus domestica*	2011	Canada	Ontario	Niagara
KAS5399	*A. arborescens*	*Malus domestica*	2011	Canada	Ontario	Niagara

KAS5400	*A. arborescens*	*Malus domestica*	2011	Canada	Ontario	Niagara
KAS5401	*A. alternata*	*Malus domestica*	2011	Canada	Ontario	Niagara
KAS5404	*A. alternata*	*Solanum lycopersicum*	2014	Canada	Ontario	Ottawa-Carleton
KAS5422	*A. alternata*	*Rubus*	2012	Canada	Ontario	Jordan Station
KAS5424	*A. arborescens*	*Rubus*	2012	Canada	Ontario	Jordan Station
KAS5427	*A. alternata*	*Solanum lycopersicum*	2014	Canada	Ontario	London
KAS5430	*A. alternata*	*Solanum lycopersicum*	2014	Canada	Ontario	London
KAS5443 (ATCC34958)	*A. alternata*	*Sorghum*		USA	Kansas	
KAS5444 (ATCC34957)	*A. alternata*	*Sorghum*		USA	Kansas	
KAS5445 (ATCC34956)	*A. alternata*	*Sorghum*		USA	Kansas	
KAS5448	*A. alternata*	*Triticum*	2013	Canada	Saskatchewan	Corrine
KAS5450	*A. alternata*	*Triticum*	2013	Canada	Saskatchewan	Corrine
KAS5453	*A. alternata*	*Avena sativa*	2013	Canada	Alberta	Edmonton
KAS5456	*A. alternata*	*Hordeum vulgare*	2013	Canada	western	
KAS5458	*A. alternata*	*Hordeum vulgare*	2013	Canada	western	
KAS5459	*A. alternata*	*Hordeum vulgare*	2013	Canada	western	
KAS5460	*A. alternata*	*Hordeum vulgare*	2013	Canada	western	
KAS5465	*A. alternata*	*Triticum*	2013	Canada	western	
KAS5478	*A. alternata*	*Triticum*	2013	Canada	western	
KAS5479	*A. alternata*	*Triticum*	2013	Canada	western	
KAS5481	*A. alternata*	*Triticum*	2013	Canada	western	
KAS5484	*A. alternata*	*Triticum*	2013	Canada	western	
KAS5485	*A. alternata*	*Triticum*	2013	Canada	western	
KAS5487	*A. alternata*	*Triticum*	2013	Canada	western	
KAS5489	*A. alternata*	*Hordeum vulgare*	2013	Canada	western	
KAS5491	*A. alternata*	*Hordeum vulgare*	2013	Canada	western	
KAS5492	*A. alternata*	*Hordeum vulgare*	2013	Canada	western	
KAS5493	*A. alternata*	*Hordeum vulgare*	2013	Canada	western	
KAS5494	*A. alternata*	*Hordeum vulgare*	2013	Canada	western	
KAS5495	*A. alternata*	*Hordeum vulgare*	2013	Canada	western	
KAS5496	*A. alternata*	*Hordeum vulgare*	2013	Canada	western	
KAS5497	*A. alternata*	*Brassica napus*	2013	Canada	western	
KAS5500	*A. alternata*	*Triticum*	2013	Canada	western	
KAS5503	*A. alternata*	*Triticum*	2013	Canada	western	
KAS5504	*A. alternata*	*Triticum*	2013	Canada	western	
KAS5508	*A. alternata*	*Triticum*	2011	Canada	Saskatchewan	Beechy
KAS5509	*A. alternata*	*Triticum*	2011	Canada	Saskatchewan	Beechy
KAS5510	*A. alternata*	*Triticum*	2011	Canada	Saskatchewan	Beechy
KAS5511	*A. alternata*	*Triticum*	2011	Canada	Saskatchewan	Beechy
KAS5512	*A. alternata*	*Triticum*	2011	Canada	Saskatchewan	Beechy
KAS5514	*A. alternata*	*Secale cereale*	2013	Canada	Quebec	
KAS5517	*A. alternata*	*Secale cereale*	2013	Canada	Quebec	
KAS5686	*A. alternata*	indoor dust	2014	Canada	Ontario	Stittsville
KAS5743	*A. alternata*	indoor dust	2015	Canada	Ontario	Ottawa
KAS5766	*A. alternata*	indoor dust	2014	Canada	Ontario	Stittsville
KAS5791	*A. alternata*	indoor dust	2014	Canada	Ontario	Stittsville
KAS5795	*A. alternata*	indoor dust	2014	Canada	Ontario	Stittsville
KAS5807	*A. alternata*	indoor dust	2014	Canada	Ontario	Stittsville
KAS5915	*A. alternata*	indoor dust	2015	Canada	British Columbia	Victoria
KAS7860	*A. alternata*	indoor dust	2015	Canada	Ontario	Ottawa
KAS7865	*A. alternata*	indoor dust	2015	Canada	Ontario	Ottawa
KAS7871	*A. alternata*	indoor dust	2015	Canada	Ontario	Ottawa
KAS7877	*A. alternata*	indoor dust	2015	Canada	Ontario	Ottawa
SLOAN15	*A. alternata*	indoor dust	2007	Canada	Saskatchewan	Regina
SLOAN220	*A. alternata*	indoor dust	2007	Canada	Saskatchewan	Regina
SLOAN2402	*A. alternata*	indoor dust	2009	South Africa	Western Cape	
SLOAN3346	*A. arborescens*	indoor dust	2009	South Africa	Western Cape	
SLOAN345	*A. arborescens*	indoor dust	2007	Canada	Saskatchewan	Regina
SLOAN66	*A. alternata*	indoor dust	2007	Canada	Saskatchewan	Regina
SLOAN7156	*A. alternata*	indoor dust	2008	Uruguay	Montevideo Dept.	
SLOAN7171	*A. arborescens*	indoor dust	2008	Uruguay	Montevideo Dept.	
SLOAN7174	*A. arborescens*	indoor dust	2008	Uruguay	Montevideo Dept.	
SLOAN7175	*A. arborescens*	indoor dust	2008	Uruguay	Montevideo Dept.	

Supplementary Table 2: Information on 39 *Alternaria* section *Alternaria* strains previously characterized in Dettman and Eggertson 2022.

Species	Taxonomy notes	Isolate name	Sampling notes	*ASA-10*	*ASA-19*	*rpb2*
A. alternata	formerly *A. longipes*	DAOMC 196737 (IMI 123 400)	Nicotiana tabacum, Malawi, 1966	OL469332	OL469375	OL469440
A. alternata		DET2015	Triticum, Canada, 2018	OL469335	OL469376	OL469427
A. alternata		DET2071	Triticum, Canada, 2018	OL469336	OL469377	OL469434
A. alternata		DET2076	Triticum, Canada, 2018	OL469337	OL469378	OL469412
A. alternata		DET2080	Triticum, Canada, 2018	OL469338	OL469379	OL469419
A. alternata		DET2123	Avena sativa, Canada, 2018	OL469339	OL469373	OL469428
A. alternata		DET2125	Avena sativa, Canada, 2018	OL469340	OL469380	OL469418
A. alternata		DET2186	Hordeum vulgare, Canada, 2018	OL469341	OL469381	OL469425
A. alternata		DET2188	Poa, Canada, 2018	OL469342	OL469394	OL469435
A. alternata		DET2196	Phalaris, Canada, 2018	OL469329	OL469382	OL469436
A. alternata		DET2201	Hymenoxys richardsonii, Canada, 1999	OL469343	OL469396	OL469431
A. alternata		DET2209	Avena sativa, Canada, 2019	OL469333	OL469383	OL469417
A. alternata		DET2219	Glycine max, Canada, 2019	OL469344	OL469384	OL469413
A. alternata		DET2231	Triticum, Canada, 2018	OL469345	OL469374	OL469414
A. alternata		DET2241	Triticum, Canada, 2018	OL469330	OL469398	OL469432
A. alternata		DET2272	Triticum, Canada, 2019	OL469346	OL469385	OL469441
A. alternata		DET2299	Triticum, Canada, 2019	OL469334	OL469386	OL469442
A. alternata		DET2351	Avena sativa, Canada, 2019	OL469347	OL469387	OL469407
A. alternata		KAS5303	Sedum, Canada, 2014	OL469348	OL469388	OL469426
A. alternata		KAS5319	Sphagnum, Canada, 2014	OL469349	OL469389	OL469437
A. alternata		KAS5380	Astilbe, Canada, 2014	OL469350	OL469390	OL469438
A. alternata		KAS5386	Calamagrostis, Canada, 2014	OL469354	OL469391	OL469443
A. alternata		KAS5425	Vaccinium angustifolium, Canada, 2012	OL469351	OL469392	OL469415
A. alternata		KAS5429	Solanum lycopersicum, Canada, 2014	OL469352	OL469393	OL469439
A. alternata		KAS5451	Avena sativa, Canada, 2013	OL469331	OL469397	OL469430
A. alternata		KAS5513	Avena sativa, Canada, 2013	OL469353	OL469395	OL469433
A. arborescens	formerly *A. gaisen*	DAOMC 234889 (EGS 90.131)	Pyrus pyrifolia, Japan, 1990	OL469358	OL469400	OL469411
A. arborescens		DET2078	Triticum, Canada, 2018	OL469355	OL469401	OL469408
A. arborescens		DET2085	Triticum, Canada, 2018	OL469356	OL469402	OL469409
A. arborescens		KAS5368	Daucus carota, Canada, 2014	OL469357	OL469403	OL469410
A. gaisen		DAOMC 231284 (EGS 90.051)	Pyrus pyrifolia, Japan, 1990	OL469362	OL469367	OL469424
A. gaisen		DAOMC 234885 (EGS 37.132)	Pyrus pyrifolia, Japan, 1984	OL469366	OL469368	OL469420
A. gaisen		DAOMC 234886 (EGS 37.133)	Pyrus pyrifolia, Japan, 1984	OL469363	OL469369	OL469423
A. gaisen		DAOMC 234887 (EGS 39.159)	Pyrus pyrifolia, Japan, 1990	OL469364	OL469370	OL469422
A. gaisen		DAOMC 234888 (EGS 90.039)	Pyrus pyrifolia, Japan, 1990	OL469365	OL469371	OL469421
A. longipes		DAOMC 231287 (EGS 30.033)	Nicotiana tabacum, USA, 1967	OL469359	OL469406	OL469444
A. longipes		KAS1274	Astronium suaveolens, Costa Rica, 1999	OL469360	OL469404	OL469445
A. longipes		KAS1275	Astronium suaveolens, Costa Rica, 1999	OL469361	OL469405	OL469446
undescribed *Alternaria* sp.		KAS6097	house dust, Canada, 2015	OL469328	OL469372	OL469416

Supplementary Table 3: Publicly available genome assembly metadata.

A) Information on 47 genome assemblies that locus sequences were extracted from and included in previous analyses.

Species	Taxonomy notes	Isolate name	Sampling notes	Accession source
A. alternata	formerly *A. tenuissima*	ANJ	soil, India	JAFLEM01
A. alternata		ATCC 11680 (BMP 0238)	Allium, USA	mycocosm.jgi.doe.gov
A. alternata		ATCC 34957	Sorghum, USA	LMXP01
A. alternata	*A. alternata* ex-type	ATCC 66891 (EGS 34–016, BMP 0269, CBS 916.96)	Arachis, India	mycocosm.jgi.doe.gov
A. alternata		B3	Ophiopogon japonicus, China	JACBJF01
A. alternata		BMP 0270 (ATCC 66982, EGS 34–039)		mycocosm.jgi.doe.gov
A. alternata	formerly *A. tenuissima* (reference)	BMP 0304 (ATCC 96828, EGS 34–015, CBS 918.96)	Dianthus, UK	mycocosm.jgi.doe.gov
A. alternata	formerly *A.*	BMP 2335 (EGS 44–159)	Citrus	mycocosm.jgi.doe.gov
A. alternata	formerly *A. citriarbusti*	BMP 2343 (EGS 46–140)	Citrus, USA	mycocosm.jgi.doe.gov
A. alternata	formerly *A. mali*	BMP 3064		mycocosm.jgi.doe.gov
A. alternata		DET2001	Triticum, Canada	JAAOQX01
A. alternata		DET2010	Triticum, Canada	JAAOQV01
A. alternata		DET2019	Triticum, Canada	JAAOQU01
A. alternata	formerly *A. tenuissima*	FERA 1082	Malus domestica	PDXA01
A. alternata	formerly *A. tenuissima*	FERA 1164	Malus domestica	PDXB01
A. alternata	apple pathotype, formerly *A. tenuissima*	FERA 1166	Malus domestica	PDXC01
A. alternata	apple pathotype	FERA 1177	Malus domestica	PDXD01
A. alternata	formerly *A. tenuissima*	FERA 24350	Pyrus pyrifolia	PDXE01
A. alternata	apple pathotype, formerly *A. tenuissima*	FERA 635	Malus domestica	PDXF01
A. alternata	formerly *A. tenuissima*	FERA 648	Pyrus	PDXG01
A. alternata	apple pathotype, formerly *A. tenuissima*	FERA 743	Malus domestica	PDXH01
A. alternata		JS-0527	Phragmites australis, South Korea	WIRD01
A. alternata		JS-1623	Abies, Korea	VZUP01
A. alternata		KAS5516	Secale cereale, Canada	JAAOQT01
A. alternata		MOD1-FUNGI5 (M1F5)	Vitus, USA	SJDQ01
A. alternata	peach pathotype	NAP07	Prunus persica, Japan	BJEP01
A. alternata		PF1	plastic debris, China	JADGIO01
A. alternata		PN1	Brassica juncea, India	VEOJ01
A. alternata		PN2	Brassica juncea, India	VEOK01
A. alternata		PPRI 21032	Allium cepa, South Africa	LSHC01
A. alternata		SRC1lrK2f	soil, USA	LXPP01
A. alternata	tangerine pathotype	Z7	Citrus tangerina	LPVP01
A. arborescens	formerly *A. mali*	BMP 3063	Malus domestica	mycocosm.jgi.doe.gov
A. arborescens		DET2008	Triticum, Canada	JAAOQW01
A. arborescens	*A. arborescens* ex-type	EGS 39-128 (CBS 102605)	Solanum lycopersicum, USA	AIIC01
A. arborescens		FERA 675	Pyrus pyrifolia	PDUP01
A. arborescens		MOD1-FUNGI6 (M1F6)	Malus domestica, USA	SJDP01
A. arborescens		NRRL 20593	Solanum lycopersicum, USA	JAACJH01
A. arborescens		RGR 97.0013	Malus domestica	PDWY01
A. arborescens		RGR 97.0016	Malus domestica	PEJP01
A. burnsii	*A. burnsii* ex-type	CBS 107.38	Cuminum cyminum, India	JAAABM01
A. gaisen	formerly *A. alternata f. sp. fragariae*	BMP 3062	Fragaria	mycocosm.jgi.doe.gov
A. gaisen	*A. gaisen* reference	EGS 90.391 (CBS 118488)	Pyrus pyrifolia, Japan	contact authors
A. gaisen	pear pathotype	FERA 650	Pyrus	PDWZ02
A. longipes	*A. longipes* reference	BMP 0313 (EGS 30–033, CBS 540.94)	Nicotiana tabacum, USA	mycocosm.jgi.doe.gov
A. longipes (*gossypina*?)	formerly *A. tangelonis*	BMP 2327 (EGS 45–080)	Citrus	mycocosm.jgi.doe.gov
A. longipes	formerly *A. alternata*, tangerine pathotype	EV-MIL-31	Citrus, China	JADAKD01

B) Information on 88 genome assemblies that locus sequences were extracted from, which were not included in previous analyses.

Species	Taxonomy notes	Isolate name	Sampling notes	Accession source
A. alternata		B2a	Allium cepa, South Africa	LSHC01
A. alternata		BC2-RLR-17S		JAJBJM01
A. alternata		C11		JAJBJN01
A. alternata		C14		JAJBJO01
A. alternata		C19		JAJBJP01
A. alternata		C20		JAJBJQ01
A. alternata		C21		JAJBJR01
A. alternata		C22		JAJBJS01
A. alternata		C24		JAJBJT01
A. alternata		C25		JAJBJU01
A. alternata		C4		JAJBJV01
A. alternata		C5		JAJBJW01
A. alternata		C6		JAJBJX01

A. alternata	accessioned as *A. alstroemeriae*	CBS 118809 (actually R-IC-06-P1) ?	indoor biome, USA	JAILXE01
A. alternata		D3		JAJBJY01
A. alternata		D4		JAJBJZ01
A. alternata		EGS_44-110		JAJBKA01
A. alternata		EGS_44-159		JAJBKB01
A. alternata		H1		JAJBKC01
A. alternata		H10		JAJBKD01
A. alternata		H11		JAJBKE01
A. alternata		H13		JAJBKF01
A. alternata		H15		JAJBKG01
A. alternata		H18		JAJBKH01
A. alternata		H19		JAJBKI01
A. alternata		H20		JAJBKJ01
A. alternata		H23		JAJBKK01
A. alternata		H3		JAJBKL01
A. alternata		hznu325	Chrysanthemum, China	JAIZPG01
A. alternata		MPI-PUGE-AT-0064	Arabidopsis thaliana, Germany	JAHBNJ01
A. alternata		X1		JAJBKM01
A. alternata		X11		JAJBKN01
A. alternata		X12		JAJBKO01
A. alternata		X15		JAJBKP01
A. alternata		X16		JAJBKQ01
A. alternata		X6		JAJBKR01
A. alternata		X7		JAJBKS01
A. alternata		X8		JAJBKT01
A. alternata		X9		JAJBKU01
A. alternata		Y1		JAJBKV01
A. alternata		Y10		JAJBKW01
A. alternata		Y12		JAJBKX01
A. alternata		Y13		JAJBKY01
A. alternata		Y14		JAJBKZ01
A. alternata		Y15		JAJBLA01
A. alternata		Y16		JAJBLB01
A. alternata		Y17		JAJBLC01
A. alternata		Y2		JAJBLD01
A. alternata		Y3		JAJBLE01
A. alternata		Y4		JAJBLF01
A. alternata		Y7		JAJBLG01
A. alternata		Y784-BC03	Actinidia chinensis, China	JAHEWI01
A. alternata		Y8		JAJBLH01
A. alternata		Z1		JAJBLJ01
A. alternata		Z10		JAJBLK01
A. alternata		Z11		JAJBLL01
A. alternata		Z12		JAJBLM01
A. alternata		Z13		JAJBLN01
A. alternata		Z14		JAJBLO01
A. alternata		Z15		JAJBLP01
A. alternata		Z2		JAJBLQ01
A. alternata		Z3		JAJBLR01
A. alternata		Z4		JAJBLS01
A. alternata		Z5		JAJBLT01
A. alternata		Z6		JAJBLU01
A. alternata		Z8		JAJBLV01
A. angustiovoidea	synonym of *A. alternata*	FKII-L2-CM-DRAB1	indoor biome, USA	JAKLNJ01
A. angustiovoidea	synonym of *A. alternata*	FKII-L8-BK-P2B	indoor biome, USA	JAKLNO01
A. gaisen		D10		JAJAUG01
A. gaisen		D11		JAJAUH01
A. gaisen		D5		JAJAUI01
A. gaisen		D7		JAJAUJ01
A. gaisen		D8		JAJAUK01
A. gaisen		D9		JAJAUL01
A. gaisen		H14		JAJAUM01
A. gaisen		H16		JAJAUN01
A. gaisen		H7		JAJAUO01
A. gaisen		H8		JAJAUP01
A. gaisen		H9		JAJAUQ01
A. gaisen		X10		JAJAUR01
A. gaisen		X13		JAJAUS01
A. gaisen		X14		JAJAUT01
A. gaisen		X2		JAJAUU01
A. gaisen		X3		JAJAUV01
A. gaisen		X5		JAJAUW01
A. longipes		CBS 540.94	Nicotiana tabacum, USA	JAHLEY01
A. postmessia	synonym of *A. alternata*	BMP 2775	Citrus, USA	JAFVLS01
A. solani (outgroup)		HWC-168-2012p		JRWV01

Supplementary Table 4: Recombination detection results.

A) Number of recombination events detected by each method

	**Detection method**						
**Dataset**	**RDP**	**GENCON**	**BootScan**	**MaxChi**	**Chimaera**	**SiScan**	**3Seq**
alternata, *ASA-10* locus	none	none	none	none	none	none	none
alternata, *ASA-19* locus	none	none	none	none	none	none	none
alternata, *rpb2* locus	none	none	none	none	none	none	none
alternata, 3 loci concatenated	none	none	none	3 events	none	1 event	4 events
arborescens, *ASA-10* locus	none	none	none	none	none	none	none
arborescens, *ASA-19* locus	none	none	none	none	none	none	none
arborescens, *rpb2* locus	none	none	none	none	none	none	none
arborescens, 3 loci concatenated	none	none	none	2 events	none	2 events	2 events

B) Corrected p-values for recombination events detected by at least two methods (3 loci concatenated)

	**Detection method**						
**Recombination event**	**RDP**	**GENCON**	**BootScan**	**MaxChi**	**Chimaera**	**SiScan**	**3Seq**
alternata, event 1	not sig	not sig	not sig	0.0060	not sig	2.10E-06	1.33E-06
alternata, event 2	not sig	not sig	not sig	0.0324	not sig	not sig	5.92E-04
alternata, event 3	not sig	not sig	not sig	0.0428	not sig	not sig	1.83E-03
arborescens, event 1	not sig	not sig	not sig	0.0329	not sig	2.63E-04	2.06E-03
arborescens, event 2	not sig	not sig	not sig	0.0313	not sig	1.44E-03	5.02E-03

C) Taxa with sequences predicted to be recombinant (inter-locus).

**Recombination event**	**List of taxa**
alternata, event 1	BMP2775, DET2003, DET2018, DET2156, DET2321, DET2352, DET2354, KAS5324, KAS7860
alternata, event 2	DET2071, DET2188, DET2196, KAS5319, KAS5380, KAS5450, KAS5481, KAS5485
alternata, event 3	DET2098, DET2191, DET2198, DET2201, DET2241, DET2332, DET2333, H20, KAS5451, KAS5478, KAS5508, KAS5509, KAS5512, KAS5513, PN1, PN2, SLOAN220
arborescens, event 1	BMP3063, DAOMC234889, EGS39_128, FERA675, KAS5398, KAS5399, KAS5400, KAS5424, M1F6, NRRL_20593, RGR97_0013, RGR97_0016
arborescens, event 2	SLOAN3346

Supplementary Table 5: Mating-type idiomorphs possessed by *A. alternata* strains

Strain	Mating-type idiomorph	Strain	Mating-type idiomorph
DET2002	MAT1-2	DET2137	MAT1-1
DET2003	MAT1-2	DET2138	MAT1-2
DET2004	MAT1-1	DET2139	MAT1-2
DET2005	MAT1-2	DET2140	MAT1-1
DET2006	MAT1-2	DET2141	MAT1-1
DET2007	MAT1-2	DET2142	MAT1-2
DET2009	MAT1-1	DET2143	MAT1-1
DET2012	MAT1-1	DET2144	MAT1-2
DET2013	MAT1-1	DET2145	MAT1-2
DET2016	MAT1-2	DET2146	MAT1-2
DET2017	MAT1-2	DET2147	MAT1-1
DET2018	MAT1-2	DET2148	MAT1-1
DET2020	MAT1-1	DET2149	MAT1-2
DET2021	MAT1-2	DET2150	MAT1-2
DET2022	MAT1-2	DET2151	MAT1-2
DET2023	MAT1-2	DET2153	MAT1-2
DET2024	MAT1-1	DET2154	MAT1-2
DET2025	MAT1-2	DET2155	MAT1-1
DET2026	MAT1-1	DET2156	MAT1-1
DET2028	MAT1-2	DET2157	MAT1-1
DET2030	MAT1-1	DET2158	MAT1-2
DET2031	MAT1-2	DET2159	MAT1-1
DET2033	MAT1-1	DET2160	MAT1-2
DET2034	MAT1-1	DET2161	MAT1-1
DET2036	MAT1-1	DET2162	MAT1-1
DET2037	MAT1-1	DET2163	MAT1-2
DET2039	MAT1-1	DET2164	MAT1-2
DET2040	MAT1-2	DET2165	MAT1-2
DET2041	MAT1-2	DET2166	MAT1-1
DET2043	MAT1-2	DET2167	MAT1-2
DET2044	MAT1-1	DET2168	MAT1-1
DET2045	MAT1-2	DET2169	MAT1-1
DET2047	MAT1-2	DET2170	MAT1-1
DET2048	MAT1-1	DET2171	MAT1-1
DET2049	MAT1-2	DET2172	MAT1-2
DET2050	MAT1-2	DET2173	MAT1-1
DET2051	MAT1-1	DET2174	MAT1-2
DET2052	MAT1-1	DET2175	MAT1-1
DET2053	MAT1-1	DET2176	MAT1-2
DET2054	MAT1-1	DET2177	MAT1-2
DET2055	MAT1-1	DET2178	MAT1-2
DET2065	MAT1-1	DET2179	MAT1-2
DET2066	MAT1-2	DET2180	MAT1-2
DET2069	MAT1-2	DET2181	MAT1-2
DET2070	MAT1-1	DET2182	MAT1-2
DET2073	MAT1-2	DET2184	MAT1-2
DET2074	MAT1-1	DET2185	MAT1-2
DET2075	MAT1-2	DET2189	MAT1-1
DET2077	MAT1-1	DET2190	MAT1-1
DET2079	MAT1-2	DET2191	MAT1-1
DET2082	MAT1-2	DET2192	MAT1-2
DET2083	MAT1-2	DET2193	MAT1-2

DET2084	MAT1-2
DET2086	MAT1-2
DET2087	MAT1-2
DET2088	MAT1-1
DET2089	MAT1-2
DET2098	MAT1-1
DET2099	MAT1-2
DET2100	MAT1-2
DET2108	MAT1-2
DET2109	MAT1-1
DET2110	MAT1-2
DET2111	MAT1-1
DET2112	MAT1-1
DET2113	MAT1-1
DET2116	MAT1-2
DET2117	MAT1-1
DET2118	MAT1-2
DET2120	MAT1-2
DET2122	MAT1-1
DET2124	MAT1-1
DET2126	MAT1-2
DET2127	MAT1-1
DET2128	MAT1-1
DET2129	MAT1-1
DET2130	MAT1-2
DET2131	MAT1-2
DET2132	MAT1-1
DET2133	MAT1-2
DET2134	MAT1-1
DET2135	MAT1-2
DET2136	MAT1-2

DET2194	MAT1-1
DET2197	MAT1-2
DET2198	MAT1-1
DET2199	MAT1-2
DET2202	MAT1-1
DET2203	MAT1-1
KAS5299	MAT1-2
KAS5306	MAT1-1
KAS5313	MAT1-2
KAS5320	MAT1-2
KAS5372	MAT1-2
KAS5394	MAT1-1
KAS5456	MAT1-2
KAS5485	MAT1-2
KAS5489	MAT1-2
KAS5497	MAT1-2
KAS5686	MAT1-2
KAS5743	MAT1-2
KAS5766	MAT1-1
KAS5791	MAT1-1
KAS5795	MAT1-1
KAS5807	MAT1-2
KAS5915	MAT1-1
KAS7860	MAT1-2
KAS7865	MAT1-1
KAS7871	MAT1-1
KAS7877	MAT1-2
SLOAN15	MAT1-2
SLOAN220	MAT1-2
SLOAN2402	MAT1-2
SLOAN66	MAT1-2
